# Supplementary material for: Co-delivery of IOX1 and doxorubicin for antibody-independent cancer chemo-immunotherapy
Source: Nat Commun. 2021 Apr 23;12:2425. doi: 10.1038/s41467-021-22407-6 (PMC8065121; doi:10.1038/s41467-021-22407-6)
Supplement: Supplementary file 1 — Supplementary Information [file 41467_2021_22407_MOESM1_ESM.pdf]

Supplementary Information for

Co-delivery of IOX1 and doxorubicin for antibody-independent cancer chemo-immunotherapy

Jing Liu[1,2ǂ], Zhihao Zhao[1,2ǂ], Nasha Qiu[1], Quan Zhou[1], Guowei Wang[1], Haiping Jiang[3], Ying Piao[1,2], Zhuxian Zhou[1,2], Jianbin Tang[1], and Youqing Shen[1,2]*

[1]Zhejiang Key Laboratory of Smart Biomaterials and Key Laboratory of Biomass Chemical Engineering of Ministry of Education, College of Chemical and Biological Engineering, Zhejiang University, Hangzhou, 310007, China. [2]Hangzhou Global Scientific and Technological Innovation Center, Hangzhou 311215, China. [3]Department of Medical Oncology, The First Affiliated Hospital, School of Medicine, Zhejiang University, Hangzhou, 310003, China.

ǂ These authors contributed equally.

* Correspondence to Youqing Shen (shenyq@zju.edu.cn)

Table of contents

Supplementary Fig. 1. The MTT assay curves of IOX1 and IOXL against cancer cells. 5
Supplementary Fig. 2. Size distribution of pegylated liposomal doxorubicin (PLD, LIBOD®) characterized by DLS. 5
Supplementary Fig. 3. The particle sizes and zeta potentials of the mixed liposomes of PLD and IOXL at different molar ratios measured by DLS. 5
Supplementary Fig. 4. The particle size distributions of IOXL in different media for different times at 4°C. 6
Supplementary Fig. 5. The particle size distributions of IPLD in different media for different times at 4°C. 6
Supplementary Fig. 6. The stability of the liposomal formulations. 6
Supplementary Fig. 7. The quantification by western blotting of P-gp expression. 7
Supplementary Fig. 8. Influence of IOX1 on P-gp ATPase activity. 7
Supplementary Fig. 9. Intracellular DOX analysis after different treatments in CT26 cells. 7
Supplementary Fig. 10. Intracellular DOX analysis after different treatments in HCT116 cells......8
Supplementary Fig. 11. P-gp activity evaluation using rhodamine 123 (Rh123). 8
Supplementary Fig. 12. Intracellular DOX analysis after different treatments in NIH-3T3 cells.....9
Supplementary Fig. 13. Construction of P-gp-knockdown or -overexpressing CT26 cells. 9
Supplementary Fig. 14. Intracellular DOX analysis after different treatments in P-gp-knockdown CT26 cells...10
Supplementary Fig. 15. Intracellular DOX analysis after different treatments in P-gp-overexpressing CT26 cells. 10

Supplementary Fig. 16. The MTT assay curves of DOX, DOX+IOX1, PLD, or IPLD against cancer cells. 11
Supplementary Fig. 17. The MTT assay curves of DOX, DOX+IOX1, PLD, IPLD, or IOX1 alone or its liposomes against NIH-3T3 or HUVEC cells. 11
Supplementary Fig. 18. The MTT assay curves of DNR, EPI, OXA or its combination with IOX1 against CT26 cells. 12
Supplementary Fig. 19. Flow cytometry analysis of CRT exposure on CT26 cells. 12
Supplementary Fig. 20. Autophagosome analysis after treatments. 12
Supplementary Fig. 21. Quantification of HMGB1 release in CT26 cells calculated from CLSM images. 13
Supplementary Fig. 22. The quantification by western blotting of p-PERK, p-eIF2α and membrane CRT expression in CT26 cells. 13
Supplementary Fig. 23. Flow cytometry analysis and the quantification of CRT exposure in HCT116 cells after treatments. 13
Supplementary Fig. 24. ATP secretion of HCT116 cells after treatments. 14
Supplementary Fig. 25. CLSM images and quantification of the nuclear HMGB1 after treatments. 14
Supplementary Fig. 26. Microscopy images of BMDCs after treatments. 14
Supplementary Fig. 27. IOX1 direct effects on DC maturation and cytokine secretion. 15
Supplementary Fig. 28. Construction of CRT-knockdown CT26 cells. 15
Supplementary Fig. 29. DC maturation in CRT-knockdown CT26 cells after treatments. 15
Supplementary Fig. 30. Membrane PD-L1 in CT26 cells after treatments analyzed by flow cytometry. 16
Supplementary Fig. 31. Membrane PD-L1 in HCT116 cells after treatments analyzed by flow cytometry. 16
Supplementary Fig. 32. PD-L1 expression in HCT116 cells after treating with IOX1. 17
Supplementary Fig. 33. Membrane PD-L1 in MCF-7 cells after treatments analyzed by flow cytometry. 17
Supplementary Fig. 34. The effect of blank liposome on PD-L1 expression in CT26 cells analyzed by flow cytometry. 17
Supplementary Fig. 35. The total PD-L1 expression of CT26 cells after treatments. 18
Supplementary Fig. 36. Construction of PD-L1-knockdown or -overexpressing CT26 cells. 18
Supplementary Fig. 37. The effects on T cell proliferation of pre-treated PD-L1-knockdown CT26 cells. 18
Supplementary Fig. 38. The effects on T cell proliferation of pre-treated PD-L1-overexpressing CT26 cells. 19
Supplementary Fig. 39. The effects on T cell activity of pre-treated PD-L1-overexpressing CT26 cells. 19
Supplementary Fig. 40. IOX1 direct effect on T cell proliferation. 19
Supplementary Fig. 41. JMJD1A downstream protein expression in JMJD1A-knockdown CT26 cells. 20
Supplementary Fig. 42. JMJD1A downstream protein expression in JMJD1A-knockdown HCT116 cells. 20
Supplementary Fig. 43. Effects of β-catenin-rescue on the JMJD1A downstream protein

expression in JMJD1A-knockdown CT26 cells. 21
Supplementary Fig. 44. Flow cytometry analysis of intracellular DOX accumulation in control or JMJD1A-knockdown CT26 cells. 21
Supplementary Fig. 45. Flow cytometry analysis of intracellular DOX accumulation in control or JMJD1A-knockdown HCT116 cells...22
Supplementary Fig. 46. Membrane PD-L1 in control or JMJD1A-knockdown CT26 cells after treatments analyzed by flow cytometry. 22
Supplementary Fig. 47. Membrane PD-L1 in control or JMJD1A-knockdown HCT116 cells after treatments analyzed by flow cytometry. 23
Supplementary Fig. 48. IOX1 effect on the P-gp and PD-L1 expression in JMJD1A-knockdown CT26 cells...23
Supplementary Fig. 49. IOX1 effect on the P-gp and PD-L1 expression in JMJD1A-knockdown HCT116 cells. 23
Supplementary Fig. 50. IOX1 treatment reduces the stability of JMJD1A protein........................24
Supplementary Fig. 51. JMJD1A and β-catenin levels in IOX1-treated CT26 cells......................25
Supplementary Fig. 52. JMJD1A and β-catenin levels in IOX1-treated HCT116 cells.................25
Supplementary Fig. 53. JMJD1A and P-gp levels in untreated murine cell lines. 26
Supplementary Fig. 54. JMJD1A and P-gp levels in untreated human cell lines...........................26
Supplementary Fig. 55. JMJD1A expression in untreated CT26 cells or T lymphocytes..............26
Supplementary Fig. 56. The blood clearance curves in BALB/c mice. 27
Supplementary Fig. 57. Biodistribution of IOX1 and DOX in s.c. CT26 tumour-bearing mice.....27
Supplementary Fig. 58. Images of the tumours from the treated s.c. 80 mm^3 CT26 tumour-bearing BALB/c mice in Figs. 5a-e. 27
Supplementary Fig. 59. Photographs of the s.c. 80 mm^3 CT26 tumour-bearing BALB/c mice treated with IPLD during the experiment. 28
Supplementary Fig. 60. The follow-up tracking of the IPLD-treated s.c. 80 mm^3 CT26 tumour-bearing BALB/c mice. 28
Supplementary Fig. 61. Blood biochemistry of the BALB/c mice after different treatments.........29
Supplementary Fig. 62. PLD treatment of s.c. JMJD1A-knockdown CT26 tumours....................29
Supplementary Fig. 63. Photographs of the s.c. 80 mm^3 JMJD1A-knockdown CT26 tumour-bearing BALB/c mice treated with PLD during the experiment. 30
Supplementary Fig. 64. JMJD1A, β-catenin, P-gp and PD-L1 expression in the IOXL-treated CT26 tumours or JMJD1A-knockdown CT26 tumours. 30
Supplementary Fig. 65. Images and the average tumour weights of the tumours from the treated s.c. 80 mm^3 CT26 tumour-bearing BALB/c nude mice in Fig. 5h. 31
Supplementary Fig. 66. The individual tumour growth curves of the s.c. 80 mm^3 CT26 tumour-bearing nude mice after treatments..31
Supplementary Fig. 67. Photographs of the s.c. 350 mm^3 CT26 tumour-bearing BALB/c mice treated with IPLD during the experiment. 32
Supplementary Fig. 68. The follow-up tracking of the IPLD-treated s.c. 350 mm^3 CT26 tumour-bearing BALB/c mice. 32
Supplementary Fig. 69. Flow cytometric quantification of intratumoural CD3$^+$ T cells in the treated s.c. 80 mm^3 CT26 tumour-bearing BALB/c mice. 33
Supplementary Fig. 70. Flow cytometric quantification of splenocytes' CD3$^+$CD8$^+$ T cells in

treated s.c. 80 mm^3 CT26 tumour-bearing BALB/c mice. 33
Supplementary Fig. 71. Flow cytometry analysis and quantification of splenocytes' Treg cells in the treated s.c. 80 mm^3 CT26 tumour-bearing BALB/c mice....................33
Supplementary Fig. 72. Flow cytometry analysis and quantification of CTLA-4 expression in intratumoural Treg cells in the treated s.c. 80 mm^3 CT26 tumour-bearing BALB/c mice..34
Supplementary Fig. 73. Flow cytometry analysis and quantification of tumour associated M2 macrophages in the treated s.c. 80 mm^3 CT26 tumour-bearing BALB/c mice. 34
Supplementary Fig. 74. Western blotting images and their quantification of PD-L1 expression in pancreas in the s.c. 80 mm^3 CT26 tumour-bearing BALB/c mice after different treatments...34
Supplementary Fig. 75. Western blotting images and their quantification of PD-L1 expression in lungs in the s.c. 80 mm^3 CT26 tumour-bearing BALB/c mice after different treatments...35
Supplementary Fig. 76. Flow cytometry analysis of intratumour $CD8^+PD\text{-}L1^+$ T cells in the s.c. 80 mm^3 CT26 tumour-bearing BALB/c mice after different treatments........35
Supplementary Fig. 77. Images of the rechallenged tumours harvested on the indicated days or at the experimental endpoint (Day 60) from the s.c. CT26 rechallenged tumour-bearing BALB/c mice in Figs. 7a-c. 35
Supplementary Fig. 78. In vivo bioluminescence imaging of the lungs in anaesthetized mice immediately after i.v. injection of ^{Luci}CT26 cells......................................36
Supplementary Fig. 79. The follow-up tracking via in vivo bioluminescence imaging and the survival of the ^{Luci}CT26-rechallenged BALB/c mice. 36
Supplementary Fig. 80. Flow cytometry analysis of splenocytes' $CD4^+CD44^+CD122^+$ T cells in the treated s.c. 80 mm^3 CT26 tumour-bearing BALB/c mice....................36
Supplementary Fig. 81. The individual tumour growth curves of the 4T1 orthotopic tumour-bearing BALB/c mice after treatments...37
Supplementary Fig. 82. The average numbers of lung metastatic nodules in the treated dual 4T1 tumour-bearing BALB/c mice. 37
Supplementary Fig. 83. Body weight changes of the mice after treatments. 37
Supplementary Fig. 84. Gating strategies for flow cytometry...38
Supplementary Table 1. Primers (5'-3') used in this study...40

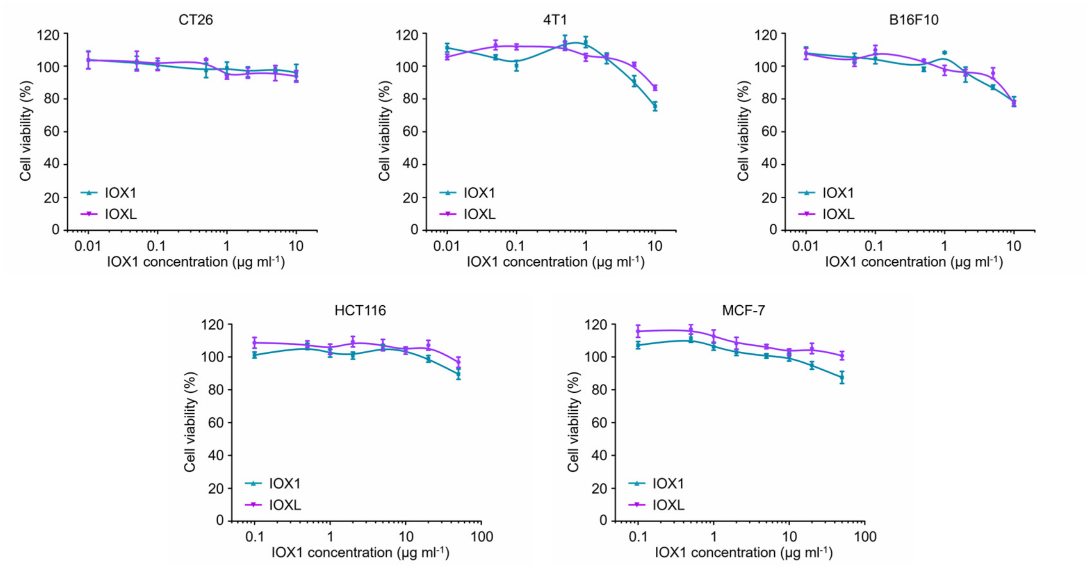

Supplementary Fig. 1. The MTT assay curves of IOX1 and IOXL against cancer cells. 48 h incubation; *n* = 3 independent experiments. Data represent mean ± SD. Source data are provided as a Source Data file.

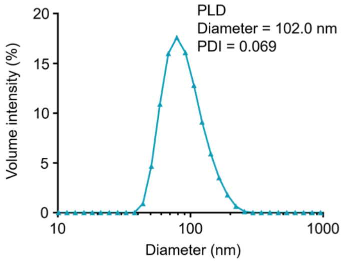

Supplementary Fig. 2. Size distribution of pegylated liposomal doxorubicin (PLD, LIBOD®) characterized by DLS. The experiment was repeated independently three times to confirm the results. Source data are provided as a Source Data file.

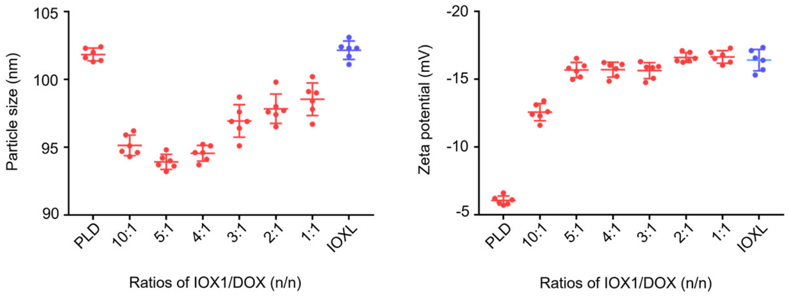

Supplementary Fig. 3. The particle sizes and zeta potentials of the mixed liposomes of PLD and IOXL at different molar ratios measured by DLS. *n* = 6 independent samples. Data represent mean ± SD. Source data are provided as a Source Data file.

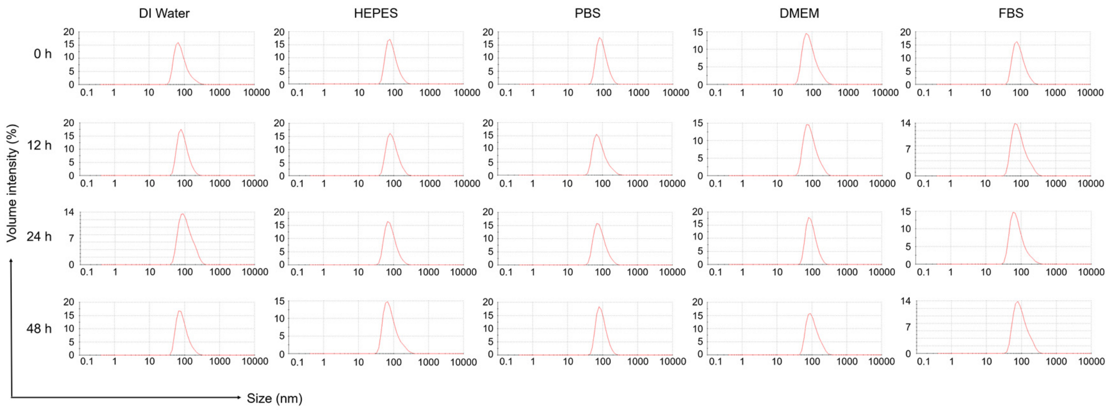

Supplementary Fig. 4. The particle size distributions of IOXL in different media for different times at 4°C. The experiment was repeated independently three times to confirm the results.

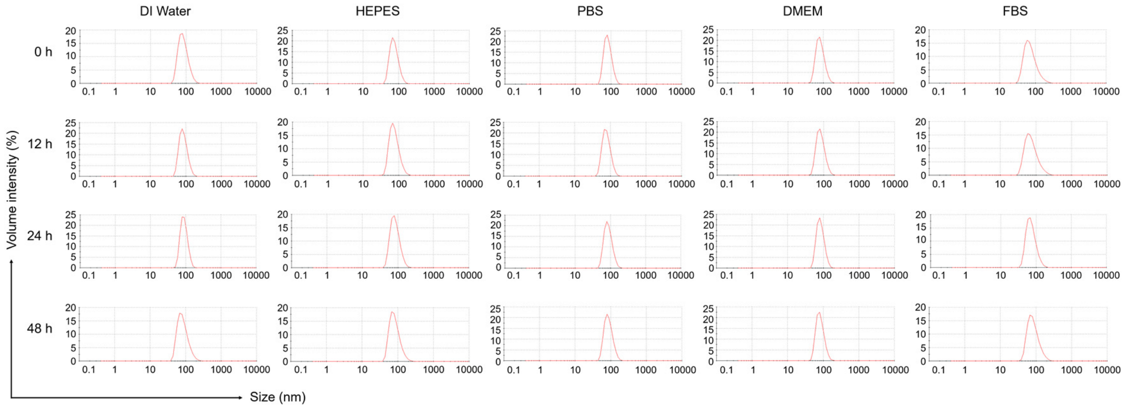

Supplementary Fig. 5. The particle size distributions of IPLD in different media for different times at 4°C. The experiment was repeated independently three times to confirm the results.

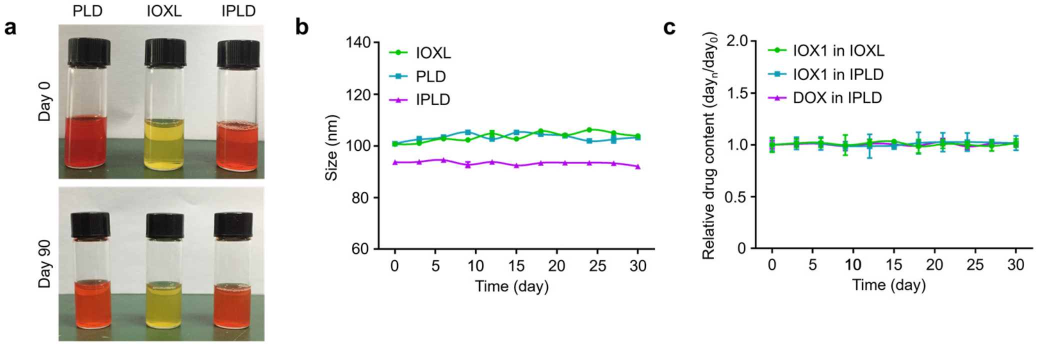

Supplementary Fig. 6. The stability of the liposomal formulations. a, The photographs of PLD, IOXL, and IPLD solutions at day 0 or after storing at 4°C for 90 days. **b,** The stability of each formulation during 30 days. **c,** The stability of IOX1 or DOX in each formulation during 30 days. n = 3 independent samples. Data represent mean ± SD. Source data are provided as a Source Data file.

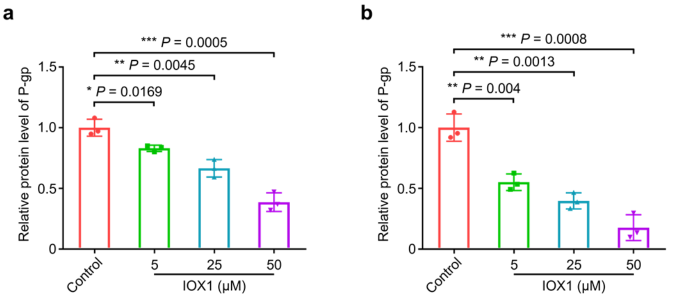

Supplementary Fig. 7. The quantification by western blotting of P-gp expression. a, CT26 or **b,** HCT116 cells were treated with IOX1 (5, 25, or 50 μM) for 24 h. $n = 3$ independent experiments. Data represent mean ± SD. Two-tailed Student's t-test. * $P < 0.05$, ** $P < 0.01$, *** $P < 0.001$. See Fig. 2c in the text for images and other conditions. Source data are provided as a Source Data file.

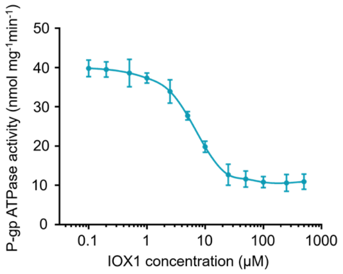

Supplementary Fig. 8. Influence of IOX1 on P-gp ATPase activity. $n = 3$ independent experiments. Data represent mean ± SD. Source data are provided as a Source Data file.

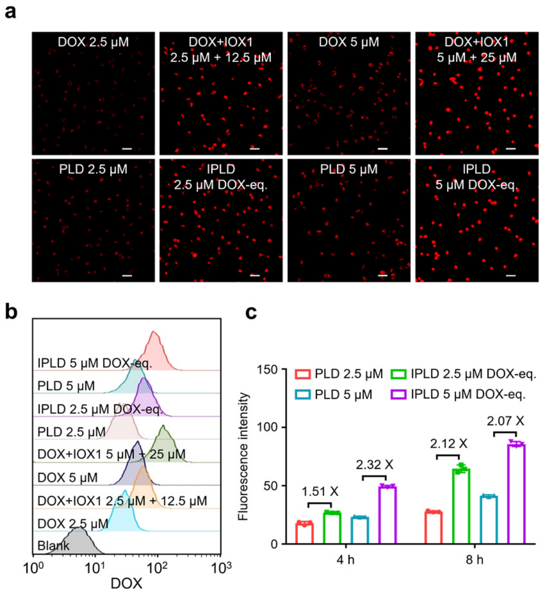

Supplementary Fig. 9. Intracellular DOX analysis after different treatments in CT26 cells. a, CLSM images and **b,** flow cytometry analysis of CT26 cells treated with DOX or its combination with IOX1 or their liposomes; 8 h culture, scale bars, 50 μm; red: DOX; The experiments were repeated independently three times to confirm the results. **c,** The intracellular DOX fluorescence intensity measured by flow cytometry of CT26 cells after different treatments for 4 h or 8 h; $n = 3$ independent experiments. Data represent mean ± SD. See Figs. 2d,e in the text for images and other conditions. Source data are provided as a Source Data file.

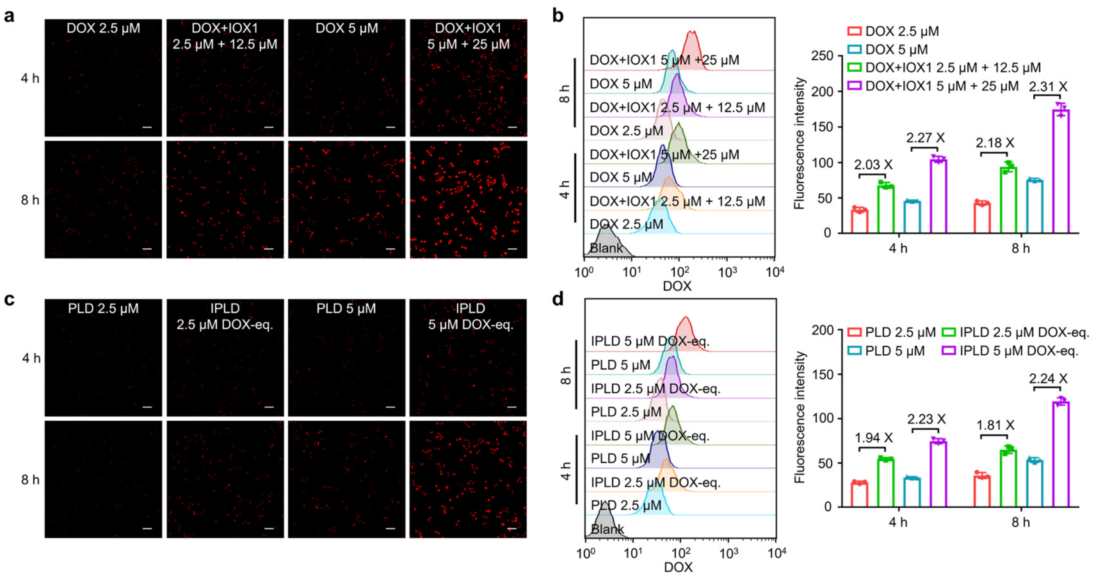

Supplementary Fig. 10. Intracellular DOX analysis after different treatments in HCT116 cells. a,c, CLSM images and **b,d,** flow cytometry analysis of HCT116 cells treated with DOX, DOX+IOX1, PLD or IPLD; 4 h or 8 h culture, scale bars, 50 μm; red: DOX. n = 3 independent experiments. Data represent mean ± SD. Source data are provided as a Source Data file.

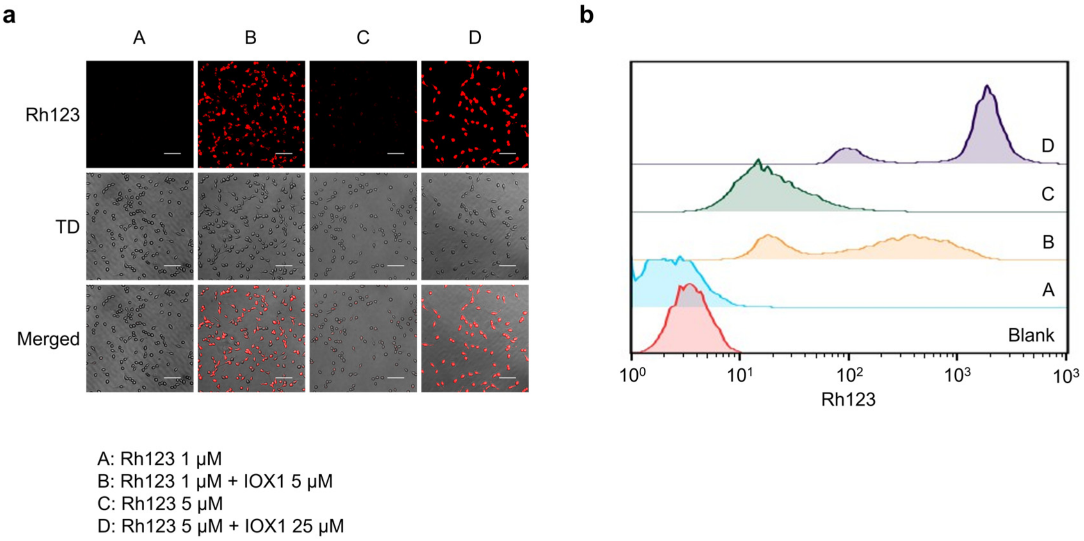

Supplementary Fig. 11. P-gp activity evaluation using rhodamine 123 (Rh123). a, CLSM images and **b,** flow cytometry analysis of intracellular Rh123 after different treatments in CT26 cells; 4 h culture, scale bars: 100 μm; red: Rh123. The experiments were repeated independently three times to confirm the results.

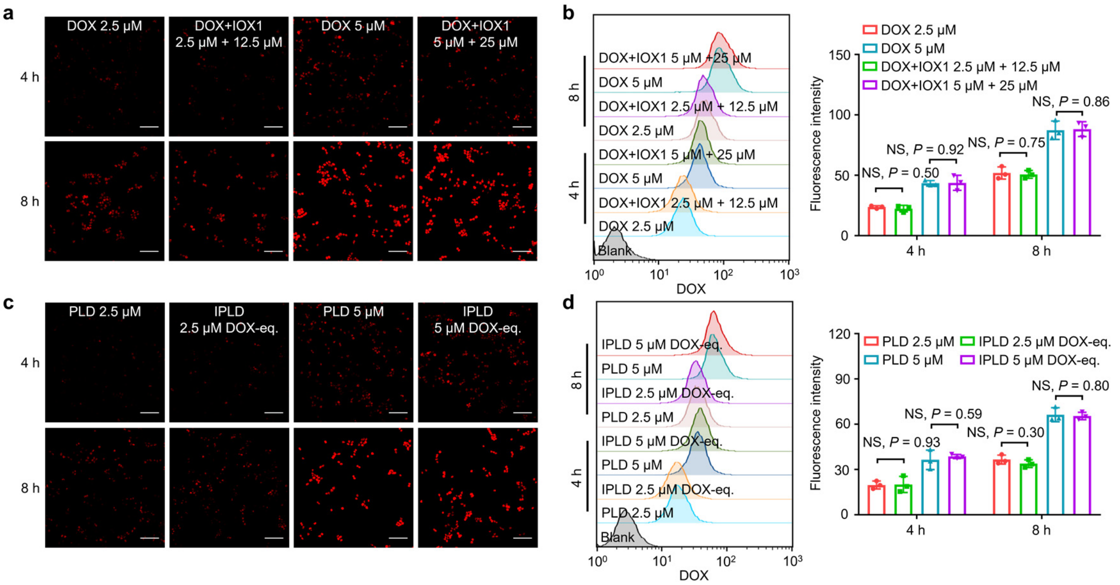

Supplementary Fig. 12. Intracellular DOX analysis after different treatments in NIH-3T3 cells. **a,c,** CLSM images and **b,d,** flow cytometry analysis of NIH-3T3 cells treated with DOX, DOX+IOX1, PLD or IPLD; 4 h or 8 h culture, scale bars, 100 μm; red: DOX. n = 3 independent experiments. Data represent mean ± SD. Two-tailed Student's t-test. NS, no significance. Source data are provided as a Source Data file.

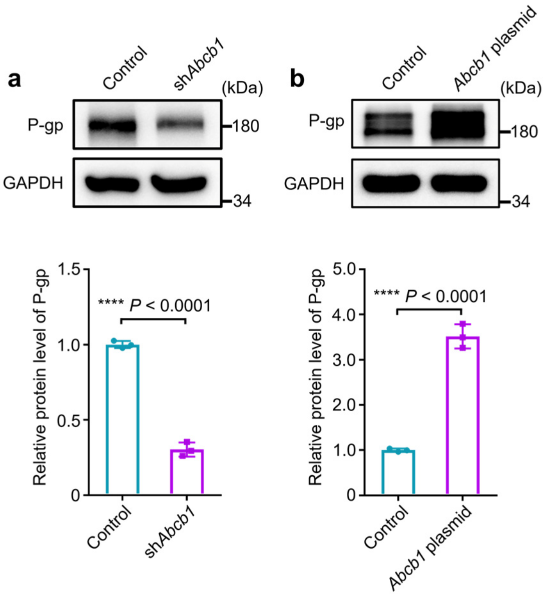

Supplementary Fig. 13. Construction of P-gp-knockdown or -overexpressing CT26 cells. Western blotting images and their quantification of P-gp expression in CT26 cells transfecting with **a,** *Abcb1* shRNA (sh*Abcb1*, 2 μg/well) or **b,** *Abcb1* plasmid (2 μg/well) for 48 h. n = 3 independent experiments. Data represent mean ± SD. Two-tailed Student's t-test. **** $P < 0.0001$. Source data are provided as a Source Data file.

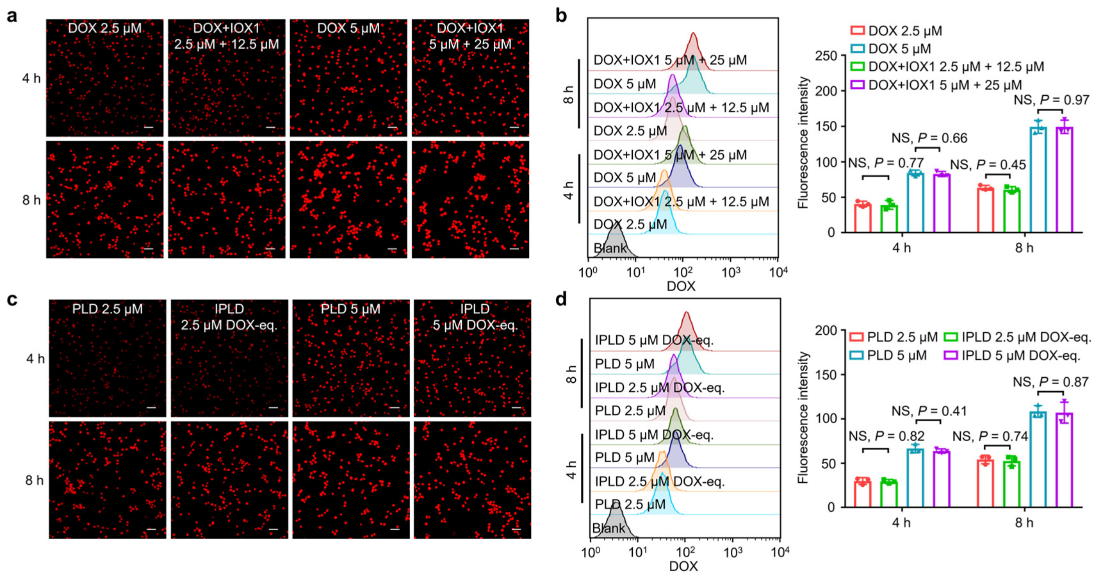

Supplementary Fig. 14. Intracellular DOX analysis after different treatments in P-gp-knockdown CT26 cells. a,c, CLSM images and **b,d,** flow cytometry analysis of P-gp-knockdown CT26 cells treated with DOX, DOX+IOX1, PLD or IPLD; 4 h or 8 h culture, scale bars, 50 μm; red: DOX. n = 3 independent experiments. Data represent mean ± SD. Two-tailed Student's t-test. NS, no significance. Source data are provided as a Source Data file.

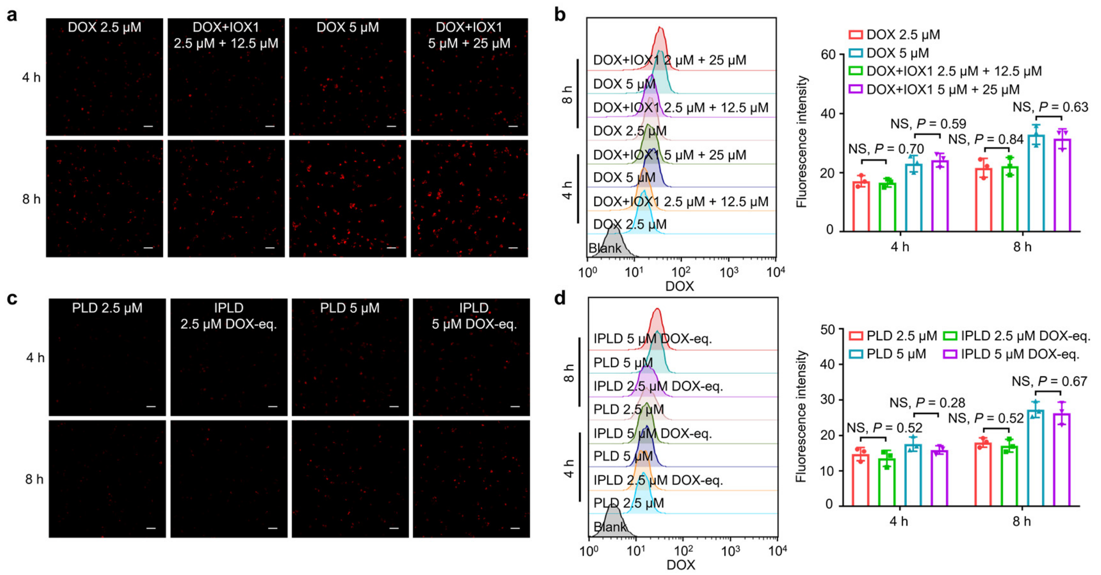

Supplementary Fig. 15. Intracellular DOX analysis after different treatments in P-gp-overexpressing CT26 cells. a,c, CLSM images and **b,d,** flow cytometry analysis of P-gp-overexpressing CT26 cells treated with DOX, DOX+IOX1, PLD or IPLD; 4 h or 8 h culture, scale bars, 50 μm; red: DOX. n = 3 independent experiments. Data represent mean ± SD. Two-tailed Student's t-test. NS, no significance. Source data are provided as a Source Data file.

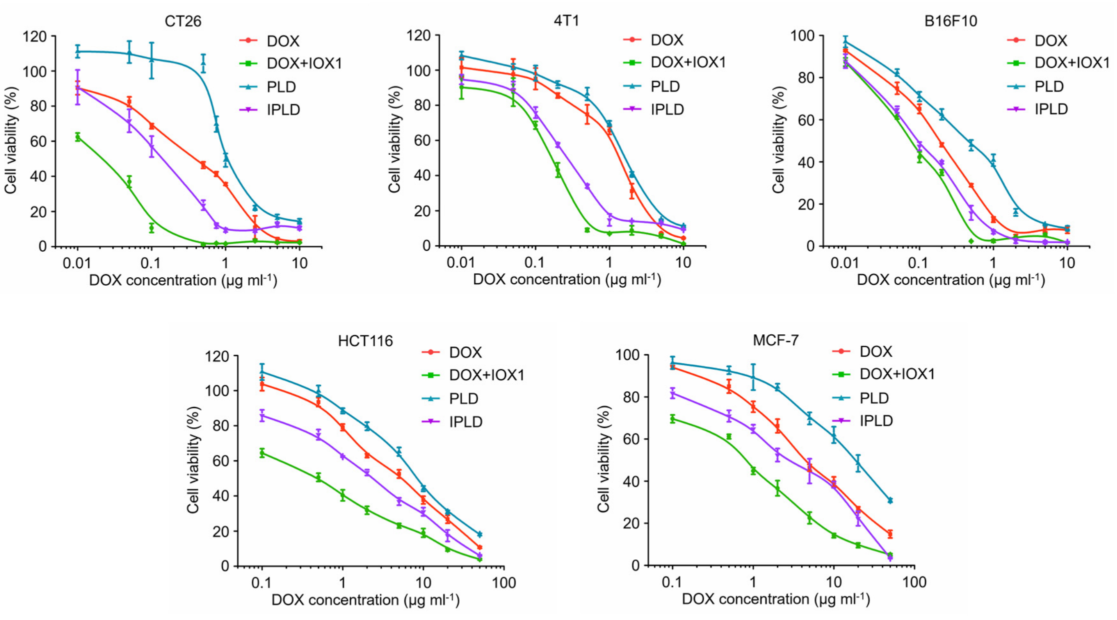

Supplementary Fig. 16. The MTT assay curves of DOX, DOX+IOX1, PLD, or IPLD against cancer cells. 48 h incubation; the molar concentration of IOX1 was kept at 5 times of that of DOX. *n* = 3 independent experiments. Data represent mean ± SD. Source data are provided as a Source Data file.

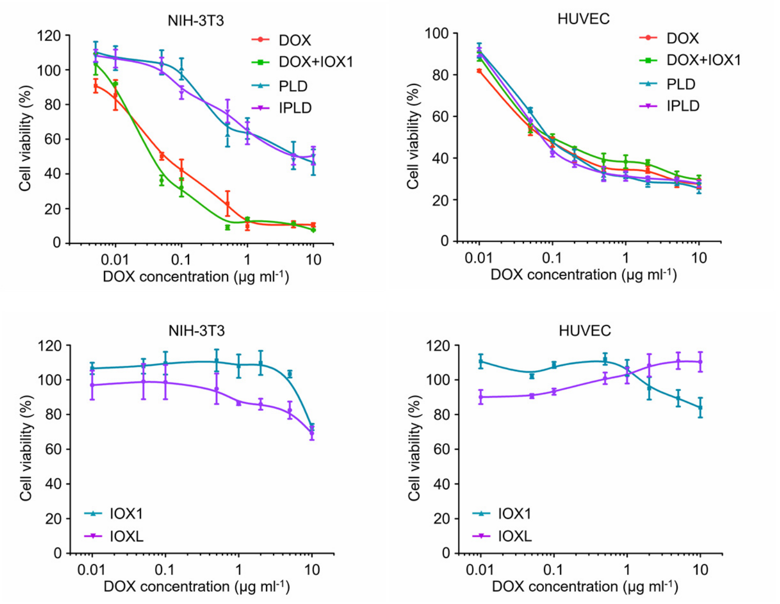

Supplementary Fig. 17. The MTT assay curves of DOX, DOX+IOX1, PLD, IPLD, or IOX1 alone or its liposomes against NIH-3T3 or HUVEC cells. 48 h incubation; the molar concentration of IOX1 was kept at 5 times of that of DOX. *n* = 3 independent experiments. Data represent mean ± SD. Source data are provided as a Source Data file.

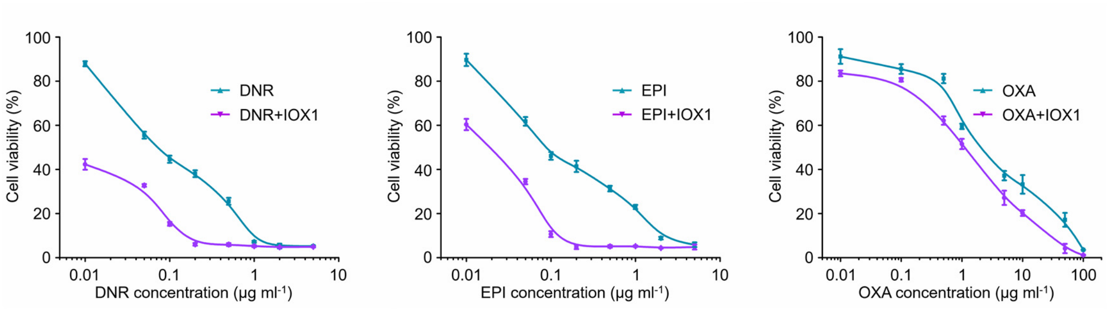

Supplementary Fig. 18. The MTT assay curves of DNR, EPI, OXA or its combination with IOX1 against CT26 cells. 48 h incubation; the molar concentration of IOX1 was kept at 5 times of that of chemotherapeutic drugs. $n = 3$ independent experiments. Data represent mean ± SD. Source data are provided as a Source Data file.

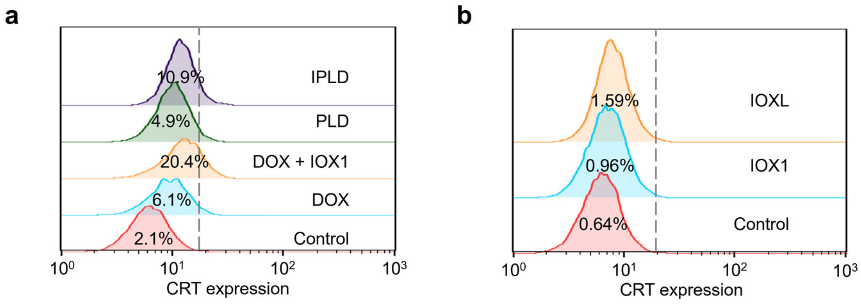

Supplementary Fig. 19. Flow cytometry analysis of CRT exposure on CT26 cells. Cells were treated for 4 h with **a,** DOX (5 μM), DOX+IOX1 (DOX, 5 μM; IOX1, 25 μM), PLD (DOX, 5 μM), IPLD (DOX, 5 μM; IOX1, 25 μM) **b,** IOX1 (25 μM) or IOXL (IOX1, 25 μM). The experiments were repeated independently three times to confirm the results.

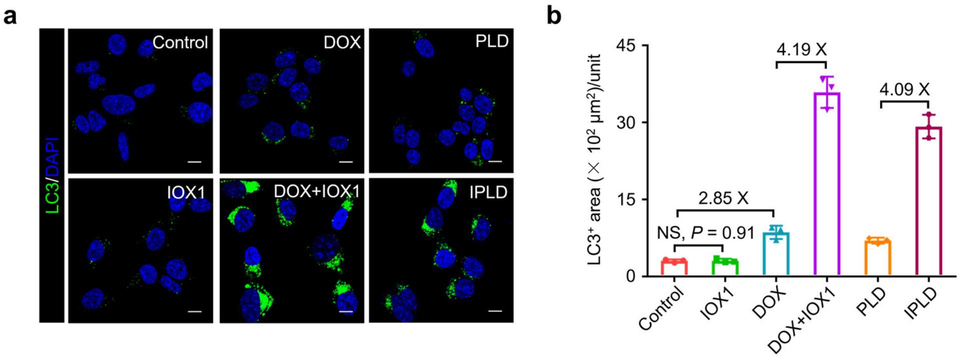

Supplementary Fig. 20. Autophagosome analysis after treatments. a, CLSM images **b,** and the quantification of the autophagosomes stained with anti-LC3 antibody (green); Cells were treated for 4 h with IOX1 (25 μM), DOX (5 μM), DOX+IOX1 (DOX, 5 μM; IOX1, 25 μM), PLD (DOX, 5 μM) or IPLD (DOX, 5 μM; IOX1, 25 μM); blue: DAPI; scale bars, 10 μm. $n = 3$ independent experiments. Data represent mean ± SD. Two-tailed Student's *t*-test. NS, no significance. Source data are provided as a Source Data file.

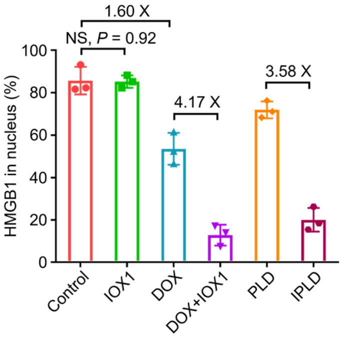

Supplementary Fig. 21. Quantification of HMGB1 release in CT26 cells calculated from CLSM images. $n = 3$ independent experiments. Data represent mean ± SD. Two-tailed Student's t-test. NS, no significance. See Fig. 2g in the text for images and conditions. Source data are provided as a Source Data file.

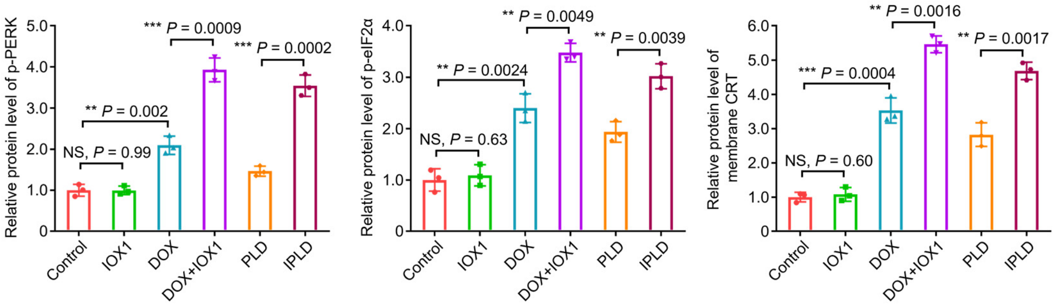

Supplementary Fig. 22. The quantification by western blotting of p-PERK, p-eIF2α and membrane CRT expression in CT26 cells. Cells were treated for 4 h with IOX1 (25 μM), DOX (5 μM), DOX+IOX1 (DOX, 5 μM; IOX1, 25 μM), PLD (DOX, 5 μM) or IPLD (DOX, 5 μM; IOX1, 25 μM). $n = 3$ independent experiments. Data represent mean ± SD. Two-tailed Student's t-test. NS, no significance; ** $P < 0.01$, *** $P < 0.001$. See Fig. 2j in the text for images and other conditions. Source data are provided as a Source Data file.

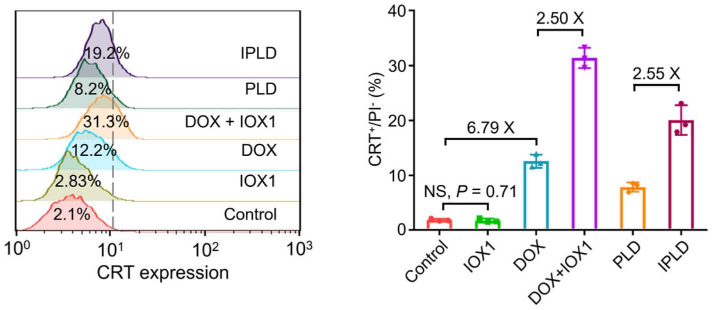

Supplementary Fig. 23. Flow cytometry analysis and the quantification of CRT exposure in HCT116 cells after treatments. Cells were treated for 4 h with IOX1 (25 μM), DOX (5 μM), DOX+IOX1 (DOX, 5 μM; IOX1, 25 μM), PLD (DOX, 5 μM) or IPLD (DOX, 5 μM; IOX1, 25 μM). $n = 3$ independent experiments. Data represent mean ± SD. Two-tailed Student's t-test. NS, no significance. Source data are provided as a Source Data file.

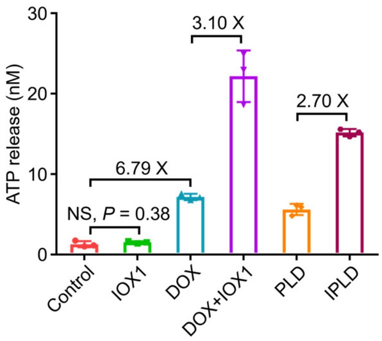

Supplementary Fig. 24. ATP secretion of HCT116 cells after treatments. Cells were treated for 4 h with IOX1 (25 μM), DOX (5 μM), DOX+IOX1 (DOX, 5 μM; IOX1, 25 μM) or PLD (DOX, 5 μM), IPLD (DOX, 5 μM; IOX1, 25 μM). n = 3 independent experiments. Data represent mean ± SD. Two-tailed Student's t-test. NS, no significance. Source data are provided as a Source Data file.

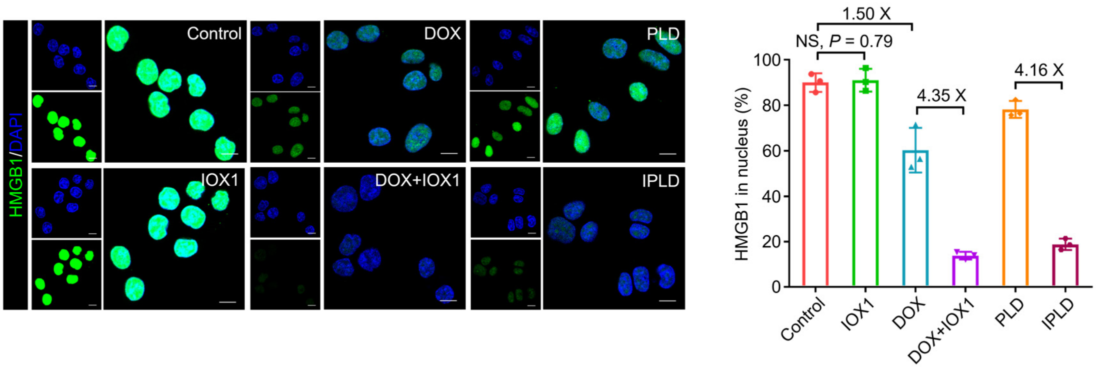

Supplementary Fig. 25. CLSM images and quantification of the nuclear HMGB1 after treatments. HCT116 cells were treated for 4 h with IOX1 (25 μM), DOX (5 μM), DOX+IOX1 (DOX, 5 μM; IOX1, 25 μM), PLD (DOX, 5 μM), IPLD (DOX, 5 μM; IOX1, 25 μM); green: HMGB1; blue: DAPI; scale bars, 10 μm. n = 3 independent experiments. Data represent mean ± SD. Two-tailed Student's t-test. NS, no significance. Source data are provided as a Source Data file.

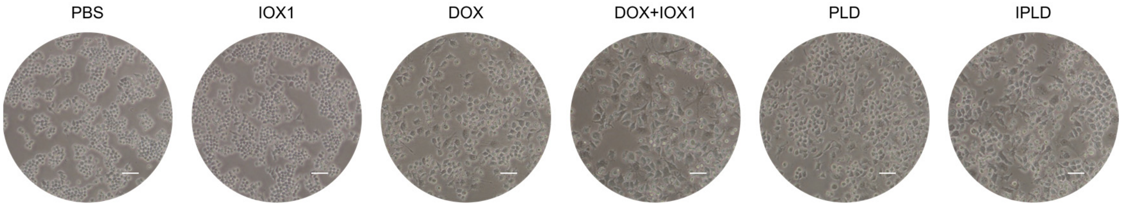

Supplementary Fig. 26. Microscopy images of BMDCs after treatments. scale bars, 50 μm. See Figs. 3a-c in the text for conditions. The experiment was repeated independently three times to confirm the results.

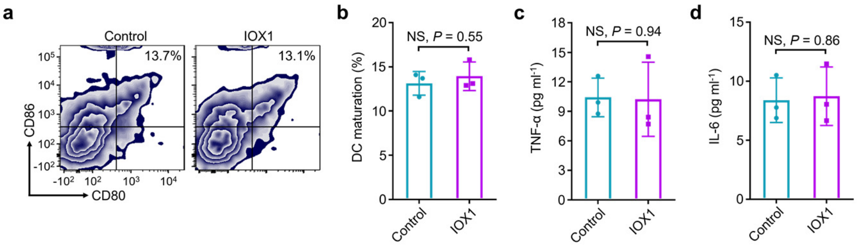

Supplementary Fig. 27. IOX1 direct effects on DC maturation and cytokine secretion. DC maturation determined by **a,** flow cytometry and **b,** quantification of the mature DCs ($CD11c^+CD80^+CD86^+$), and their secretion of **c,** TNF-α and **d,** IL-6 in supernatants after treating for 24 h with IOX1 (25 μM) without CT26 cells. n = 3 independent experiments. Data represent mean ± SD. Two-tailed Student's t-test. NS, no significance. Source data are provided as a Source Data file.

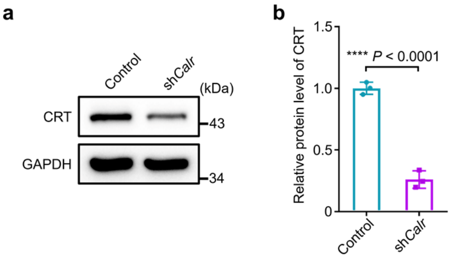

Supplementary Fig. 28. Construction of CRT-knockdown CT26 cells. a, Western blotting images and **b,** their quantification of CRT expression in CT26 cells after transfecting for 48 h with CRT shRNA (sh*Calr*, 2 μg/well). n = 3 independent experiments. Data represent mean ± SD. Two-tailed Student's t-test. **** $P < 0.0001$. Source data are provided as a Source Data file.

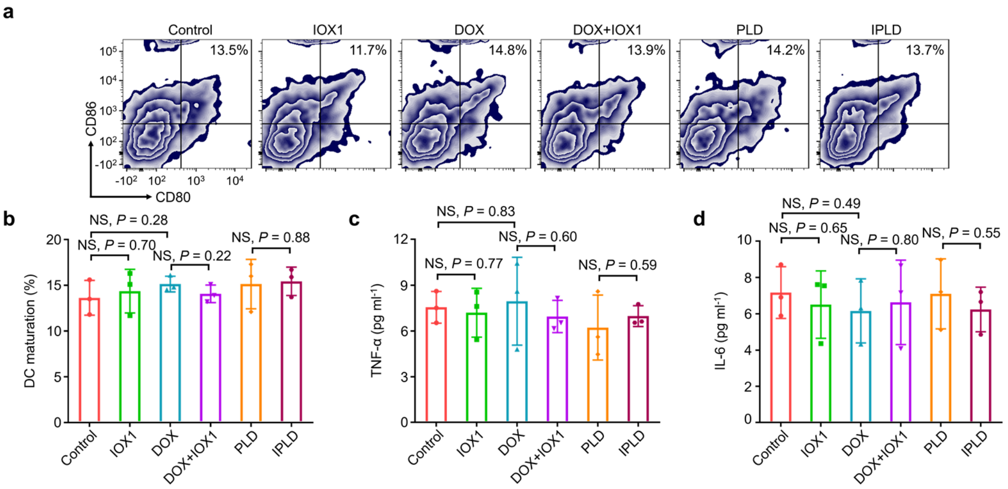

Supplementary Fig. 29. DC maturation in CRT-knockdown CT26 cells after treatments. DC maturation determined by **a,** flow cytometry and **b,** quantification of the mature DCs ($CD11c^+CD80^+CD86^+$), and their secretion of **c,** TNF-α and **d,** IL-6 in supernatants after co-culturing with CRT-knockdown CT26 cells pre-treated with IOX1 (25 μM), DOX (5 μM), DOX+IOX1 (DOX, 5 μM; IOX1, 25 μM), PLD (DOX, 5 μM) or IPLD (DOX, 5 μM; IOX1, 25 μM). n = 3 independent experiments. Data represent mean ± SD. Two-tailed Student's t-test. NS, no significance. Source data are provided as a Source Data file.

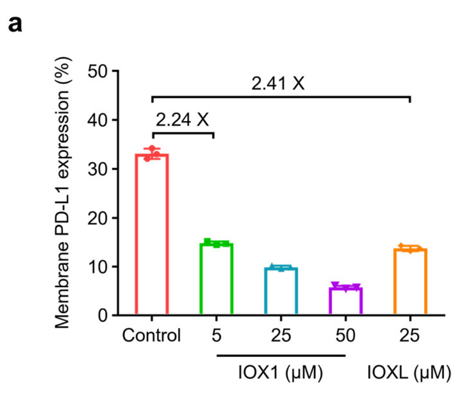

Supplementary Fig. 30. Membrane PD-L1 in CT26 cells after treatments analyzed by flow cytometry. Cells were treated for 24 h with **a,** IOX1 (5, 25, or 50 μM), IOXL (IOX1, 25 μM), **b,** DOX (5 μM), DOX+IOX1 (DOX, 5 μM; IOX1, 25 μM), PLD (DOX, 5 μM) or IPLD (DOX, 5 μM; IOX1, 25 μM). *n* = 3 independent experiments. Data represent mean ± SD. Two-tailed Student's *t*-test. See Fig. 3d in the text for histograms and other conditions. Source data are provided as a Source Data file.

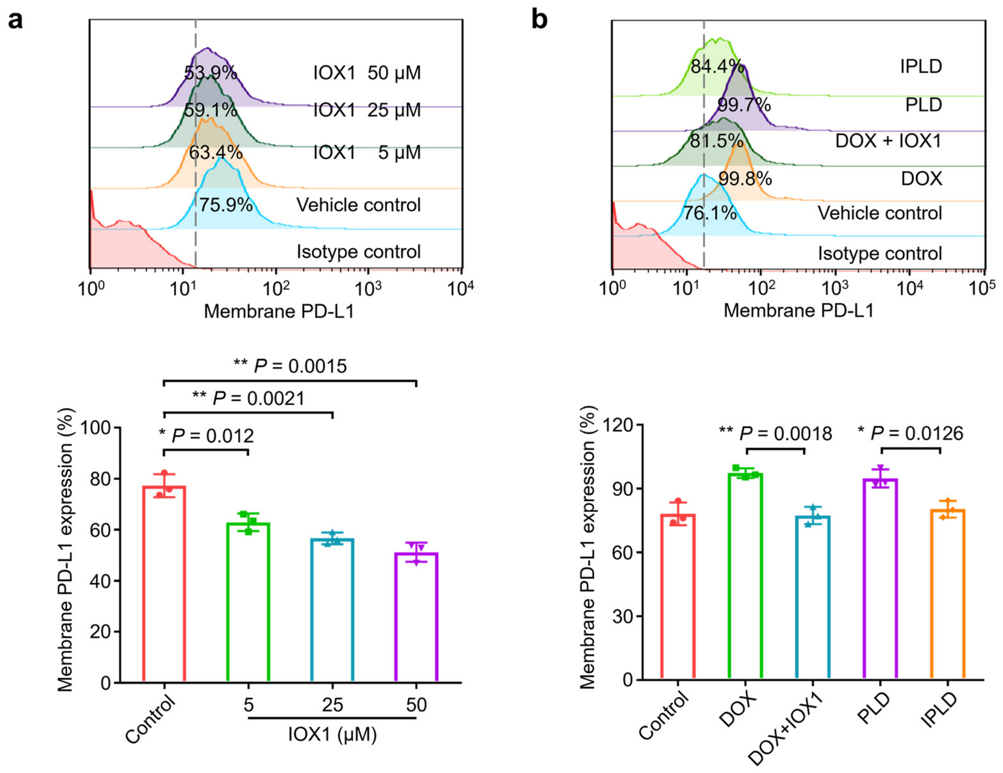

Supplementary Fig. 31. Membrane PD-L1 in HCT116 cells after treatments analyzed by flow cytometry. HCT116 cells were treated with **a,** IOX1 (5, 25, or 50 μM), **b,** DOX (5 μM), DOX+IOX1 (DOX, 5 μM; IOX1, 25 μM), PLD (DOX, 5 μM) or IPLD (DOX, 5 μM; IOX1, 25 μM) for 24 h. *n* = 3 independent experiments. Data represent mean ± SD. Two-tailed Student's *t*-test. * $P < 0.05$, ** $P < 0.01$. Source data are provided as a Source Data file.

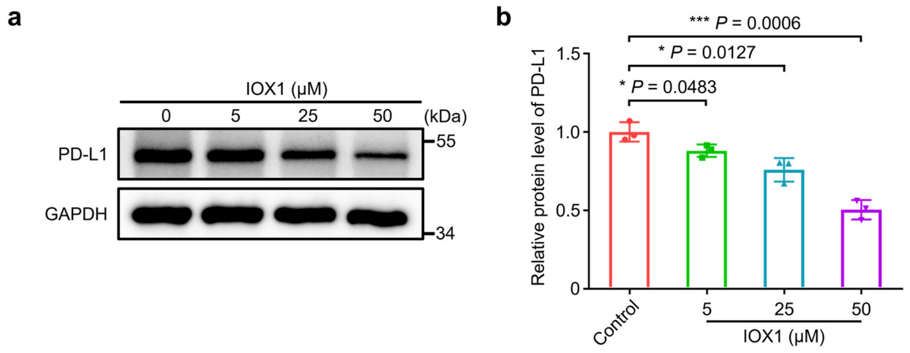

Supplementary Fig. 32. PD-L1 expression in HCT116 cells after treating with IOX1. a, Western blotting images and **b,** their quantification; HCT116 cells were treated with IOX1 (5, 25, or 50 μM) for 24 h. $n = 3$ independent experiments. Data represent mean ± SD. Two-tailed Student's t-test. * $P < 0.05$, *** $P < 0.001$. Source data are provided as a Source Data file.

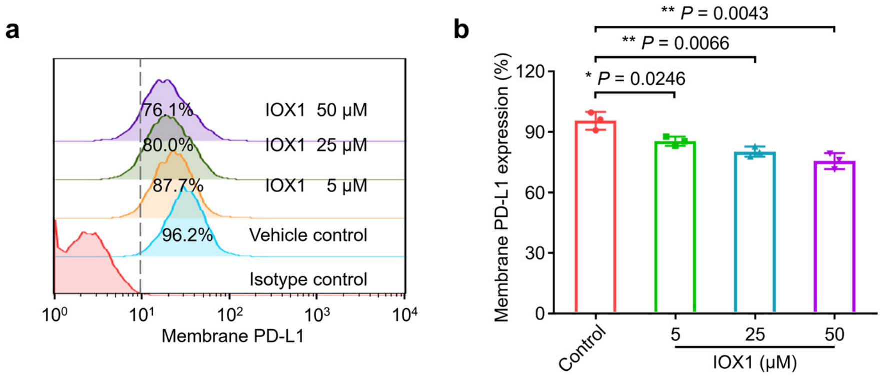

Supplementary Fig. 33. Membrane PD-L1 in MCF-7 cells after treatments analyzed by flow cytometry. a, Flow cytometry analysis and **b,** quantification; MCF-7 cells were treated with IOX1 (5, 25, or 50 μM) for 24 h. $n = 3$ independent experiments. Data represent mean ± SD. Two-tailed Student's t-test. * $P < 0.05$, ** $P < 0.01$. Source data are provided as a Source Data file.

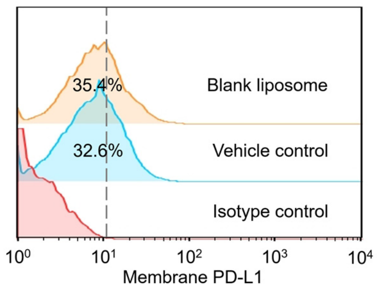

Supplementary Fig. 34. The effect of blank liposome on PD-L1 expression in CT26 cells analyzed by flow cytometry. Cells were treated with blank liposomes equivalent to the lipid concentration of 25 μM IOXL for 24 h. The experiment was repeated independently three times to confirm the results.

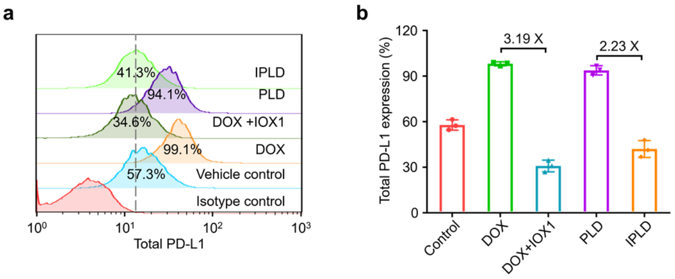

Supplementary Fig. 35. The total PD-L1 expression of CT26 cells after treatments. a, Flow cytometry analysis and **b,** quantification; Cells were treated with DOX (5 µM), DOX+IOX1 (DOX, 5 µM; IOX1, 25 µM), PLD (DOX, 5 µM) or IPLD (DOX, 5 µM; IOX1, 25 µM) for 24 h. n = 3 independent experiments. Data represent mean ± SD. Source data are provided as a Source Data file.

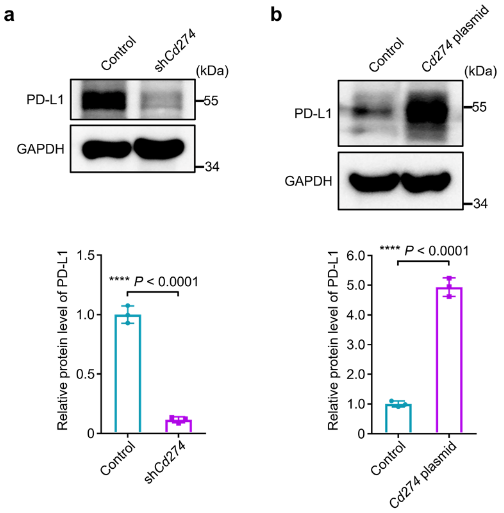

Supplementary Fig. 36. Construction of PD-L1-knockdown or -overexpressing CT26 cells. Western blotting images and their quantification of PD-L1 expression in CT26 cells transfecting with **a,** PD-L1 shRNA (sh*Cd274*, 2 µg/well) or **b,** *Cd274* plasmid (2 µg/well) for 48 h. n = 3 independent experiments. Data represent mean ± SD. Two-tailed Student's t-test. **** $P < 0.0001$. Source data are provided as a Source Data file.

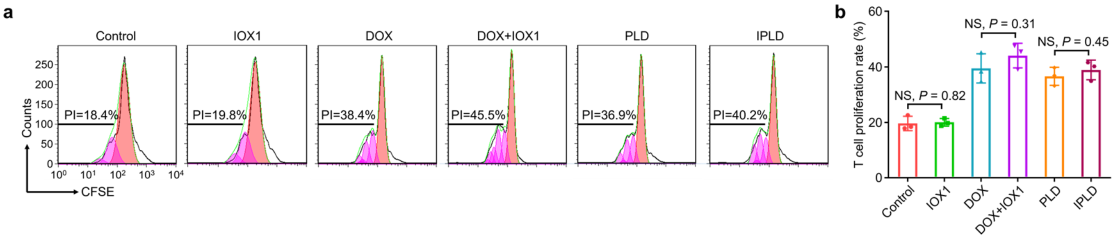

Supplementary Fig. 37. The effects on T cell proliferation of pre-treated PD-L1-knockdown CT26 cells. a, Flow cytometric profiles and **b,** their calculated proliferation indices (PIs) of T cells after co-culturing with PD-L1-knockdown CT26 cells pre-treated with IOX1, DOX, their combination, PLD, or IPLD; IOX1 dose, 25 µM; DOX dose, 5 µM; 24 h; n = 3 independent experiments. Data represent mean ± SD. Two-tailed Student's t-test. NS, no significance. Source data are provided as a Source Data file.

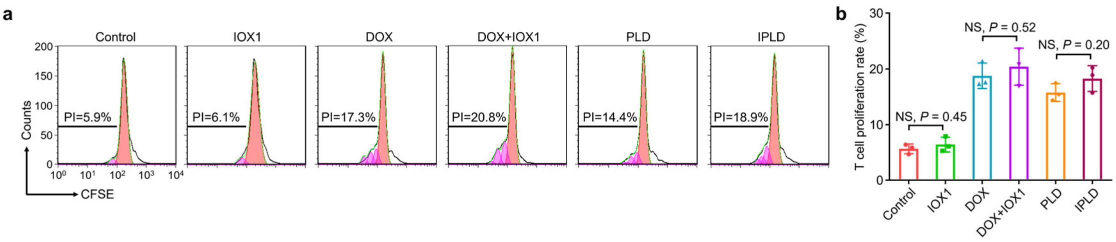

Supplementary Fig. 38. The effects on T cell proliferation of pre-treated PD-L1-overexpressing CT26 cells. a, Flow cytometric profiles and **b,** their calculated proliferation indices (PIs) of T cells after co-culturing with PD-L1-overexpressing CT26 cells pre-treated with IOX1, DOX, their combination, PLD, or IPLD; IOX1 dose, 25 μM; DOX dose, 5 μM; 24 h; n = 3 independent experiments. Data represent mean ± SD. Two-tailed Student's t-test. NS, no significance. Source data are provided as a Source Data file.

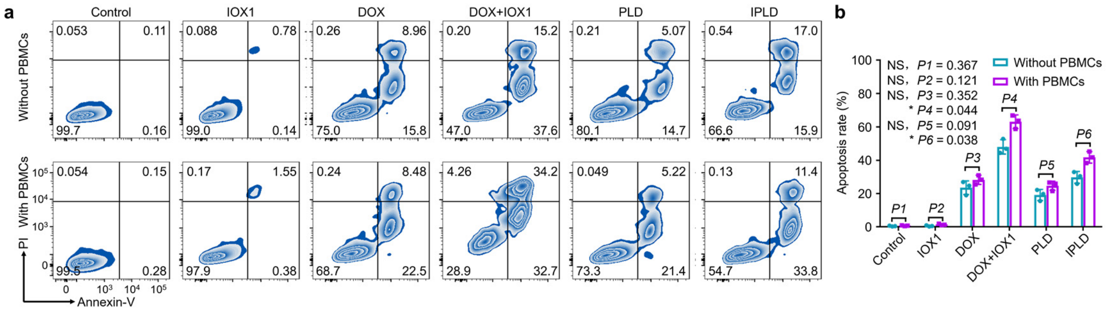

Supplementary Fig. 39. The effects on T cell activity of pre-treated PD-L1-overexpressing CT26 cells. a, Flow cytometric analysis and **b,** quantification of the apoptotic PD-L1-overexpressing CT26 cells pre-treated with IOX1, DOX, their combination, PLD, or IPLD for 24 h with or without PBMCs co-culturing; IOX1 dose, 25 μM; DOX dose, 5 μM; n = 3 independent experiments. Data represent mean ± SD. Two-tailed Student's t-test. NS, no significance; * $P < 0.05$. Source data are provided as a Source Data file.

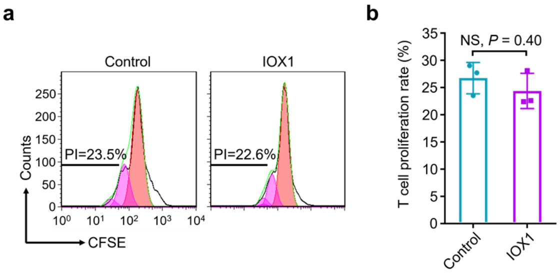

Supplementary Fig. 40. IOX1 direct effect on T cell proliferation. a, Flow cytometric profiles and **b,** their calculated proliferation indices (PIs) of T cells after treating with IOX1 (25 μM) for 72 h without CT26 cells. n = 3 independent experiments. Data represent mean ± SD. Two-tailed Student's t-test. NS, no significance. Source data are provided as a Source Data file.

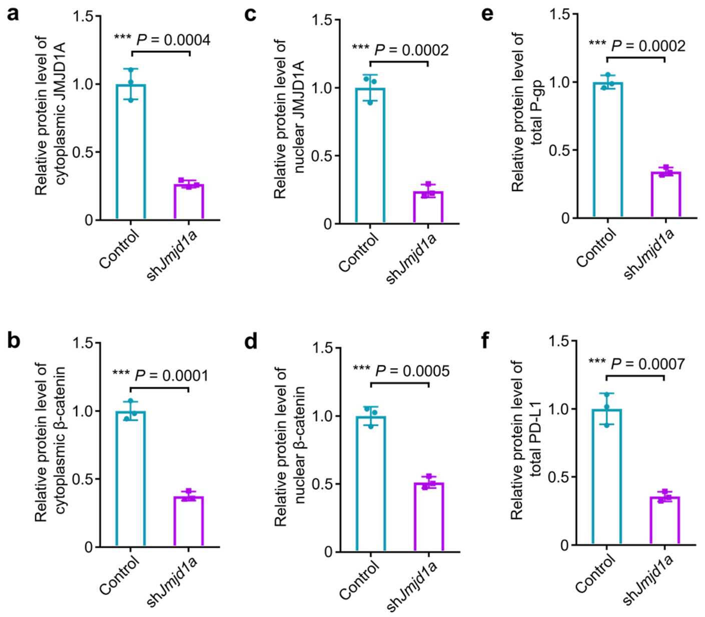

Supplementary Fig. 41. JMJD1A downstream protein expression in JMJD1A-knockdown CT26 cells. Quantification by western blotting of **a,** cytoplasmic JMJD1A, **b,** cytoplasmic β-catenin, **c,** nuclear JMJD1A, **d,** nuclear β-catenin, **e,** total P-gp and **f,** total PD-L1 in CT26 cells at 48 h after transfecting with sh*Jmjd1a* (2 μg/well). $n = 3$ independent experiments. Data represent mean ± SD. Two-tailed Student's *t*-test. *** $P < 0.001$. See Fig. 4b in the text for images and other conditions. Source data are provided as a Source Data file.

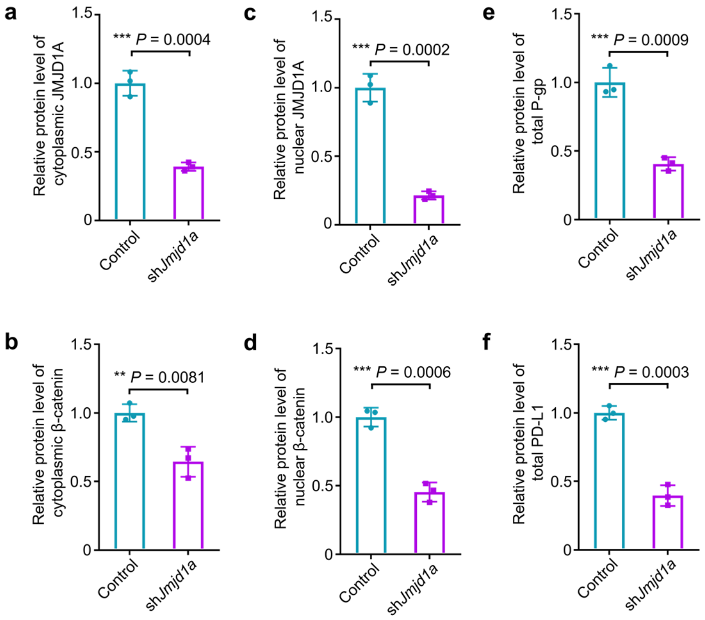

Supplementary Fig. 42. JMJD1A downstream protein expression in JMJD1A-knockdown HCT116 cells. Quantification by western blotting of **a,** cytoplasmic JMJD1A, **b,** cytoplasmic β-catenin, **c,** nuclear JMJD1A, **d,** nuclear β-catenin, **e,** total P-gp and **f,** total PD-L1 in HCT116 cells after transfecting for 48 h with sh*Jmjd1a* (2 μg/well). $n = 3$ independent experiments. Data represent mean ± SD. Two-tailed Student's *t*-test. ** $P < 0.01$, *** $P < 0.001$. See Fig. 4c in the text for images and other conditions. Source data are provided as a Source Data file.

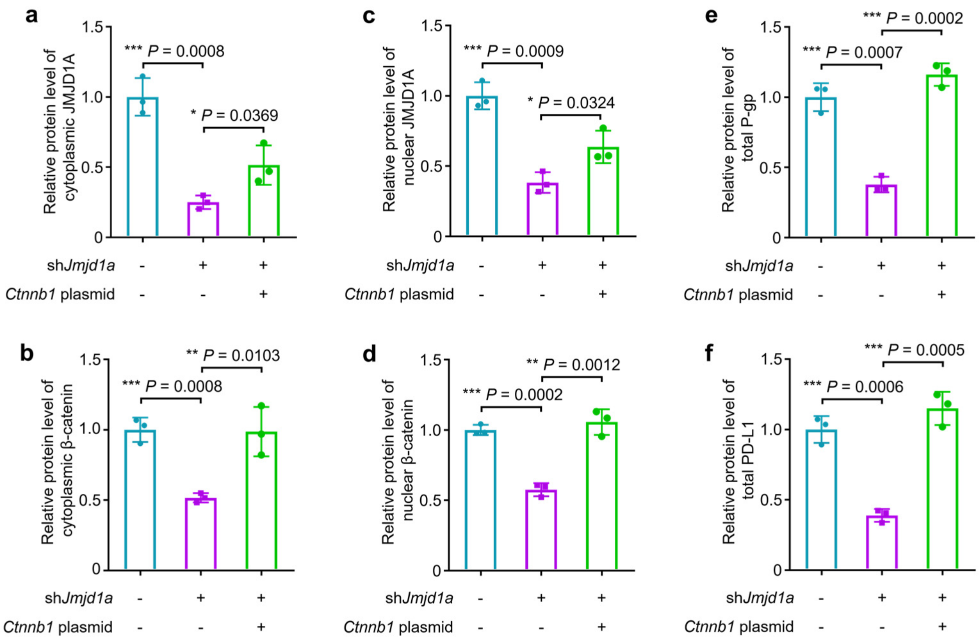

Supplementary Fig. 43. Effects of β-catenin-rescue on the JMJD1A downstream protein expression in JMJD1A-knockdown CT26 cells. Quantification by western blotting of **a,** cytoplasmic JMJD1A, **b,** cytoplasmic β-catenin, **c,** nuclear JMJD1A, **d,** nuclear β-catenin, **e,** total P-gp and **f,** total PD-L1. CT26 cells were transfected with sh*Jmjd1a* (2 μg/well) for 48 h and then with *Ctnnb1* plasmid (2 μg/well) for 24 h. $n = 3$ independent experiments. Data represent mean ± SD. Two-tailed Student's *t*-test. * $P < 0.05$, ** $P < 0.01$, *** $P < 0.001$. See Fig. 4f in the text for images and other conditions. Source data are provided as a Source Data file.

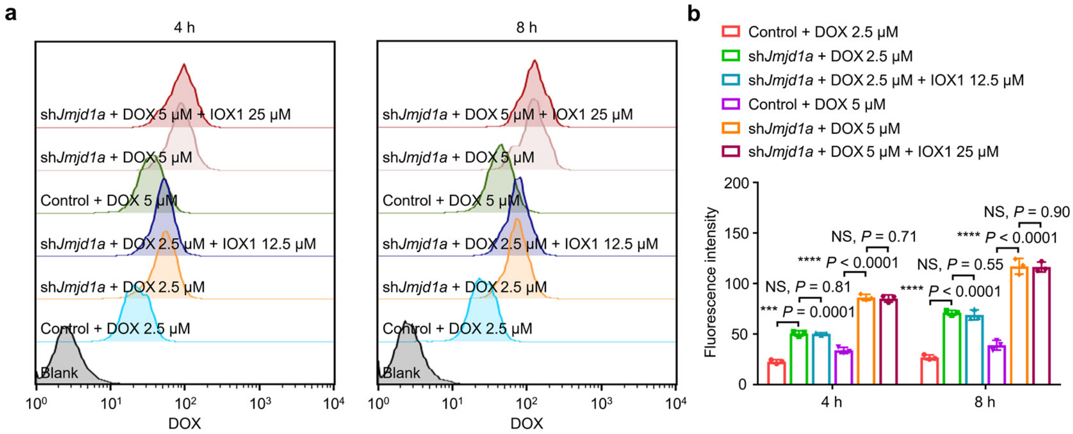

Supplementary Fig. 44. Flow cytometry analysis of intracellular DOX accumulation in control or JMJD1A-knockdown CT26 cells. a, Flow cytometry analysis and **b,** quantification; 4 h or 8 h treatment with DOX; the sh*Jmjd1a* +DOX groups are the cells first transfected with sh*Jmjd1a* for 48 h and then cultured with DOX or DOX+IOX1 at the indicated doses for 4 or 8 h. $n = 3$ independent experiments. Data represent mean ± SD. Two-tailed Student's *t*-test. NS, no significance; *** $P < 0.001$, **** $P < 0.0001$. Source data are provided as a Source Data file.

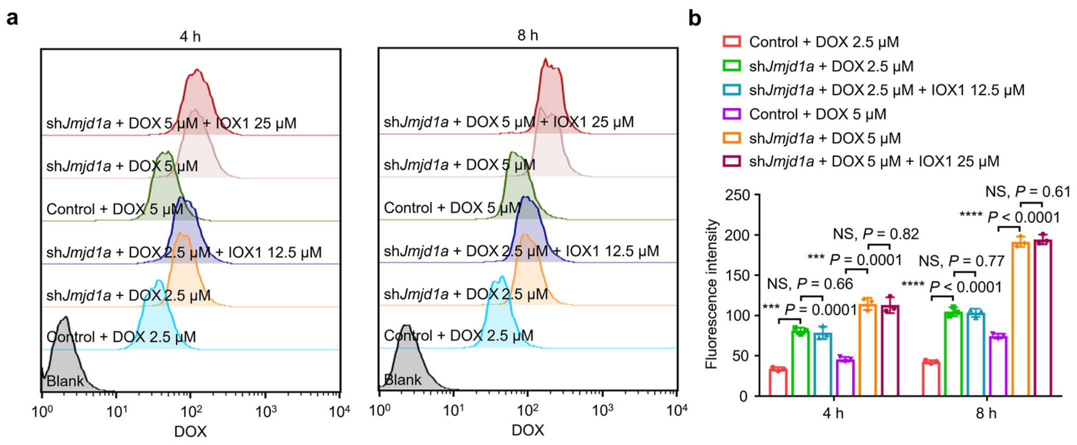

Supplementary Fig. 45. Flow cytometry analysis of intracellular DOX accumulation in control or JMJD1A-knockdown HCT116 cells. a, Flow cytometry analysis and **b,** quantification; 4 h or 8 h treatment with DOX; the sh*Jmjd1a* +DOX groups are the cells first transfected with sh*Jmjd1a* for 48 h and then cultured with DOX or DOX+IOX1 at the indicated doses for 4 or 8 h. $n = 3$ independent experiments. Data represent mean ± SD. Two-tailed Student's t-test. NS, no significance; *** $P < 0.001$, **** $P < 0.0001$. Source data are provided as a Source Data file.

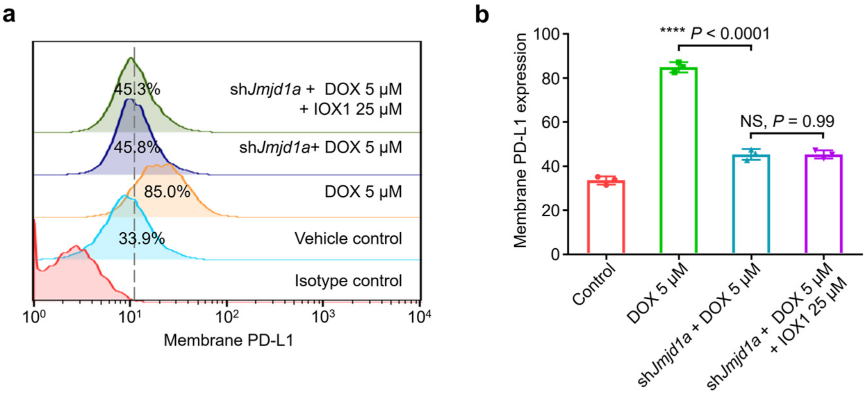

Supplementary Fig. 46. Membrane PD-L1 in control or JMJD1A-knockdown CT26 cells after treatments analyzed by flow cytometry. a, Flow cytometry analysis and **b,** quantification; Control or JMJD1A-knockdown CT26 cells were treated with DOX (5 μM) or DOX+IOX1 (DOX, 5 μM; IOX1, 25 μM) for 24 h. $n = 3$ independent experiments. Data represent mean ± SD. Two-tailed Student's t-test. NS, no significance; **** $P < 0.0001$. Source data are provided as a Source Data file.

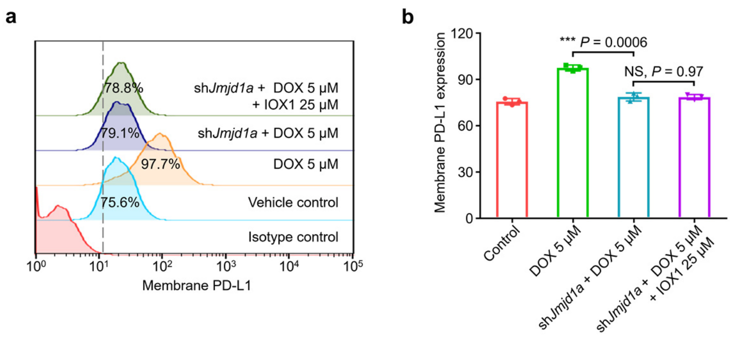

Supplementary Fig. 47. Membrane PD-L1 in control or JMJD1A-knockdown HCT116 cells after treatments analyzed by flow cytometry. a, Flow cytometry analysis and **b,** quantification; Control or JMJD1A-knockdown HCT116 cells were treated with DOX (5 μM) or DOX+IOX1 (DOX, 5 μM; IOX1, 25 μM) for 24 h. $n = 3$ independent experiments. Data represent mean ± SD. Two-tailed Student's *t*-test. NS, no significance; *** $P < 0.001$. Source data are provided as a Source Data file.

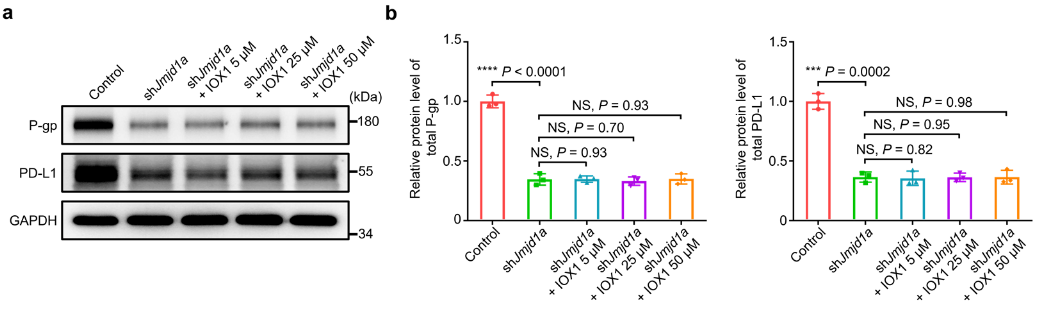

Supplementary Fig. 48. IOX1 effect on the P-gp and PD-L1 expression in JMJD1A-knockdown CT26 cells. a, Western blotting images and **b,** their quantification; JMJD1A-knockdown CT26 cells were treated with IOX1 (5, 25, or 50 μM) for 24 h. $n = 3$ independent experiments. Data represent mean ± SD. Two-tailed Student's *t*-test. NS, no significance; *** $P < 0.001$, **** $P < 0.0001$. Source data are provided as a Source Data file.

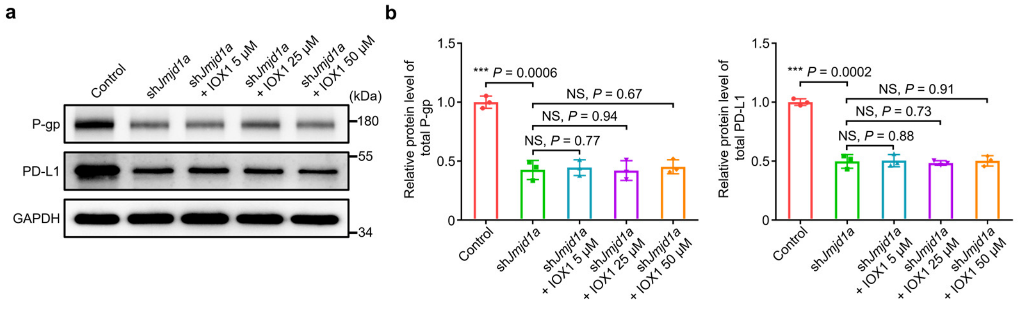

Supplementary Fig. 49. IOX1 effect on the P-gp and PD-L1 expression in JMJD1A-knockdown HCT116 cells. a, Western blotting images and **b,** their quantification; JMJD1A-knockdown HCT116 cells were treated with IOX1 (5, 25, or 50 μM) for 24 h. $n = 3$ independent experiments. Data represent mean ± SD. Two-tailed Student's *t*-test. NS, no significance; *** $P < 0.001$. Source data are provided as a Source Data file.

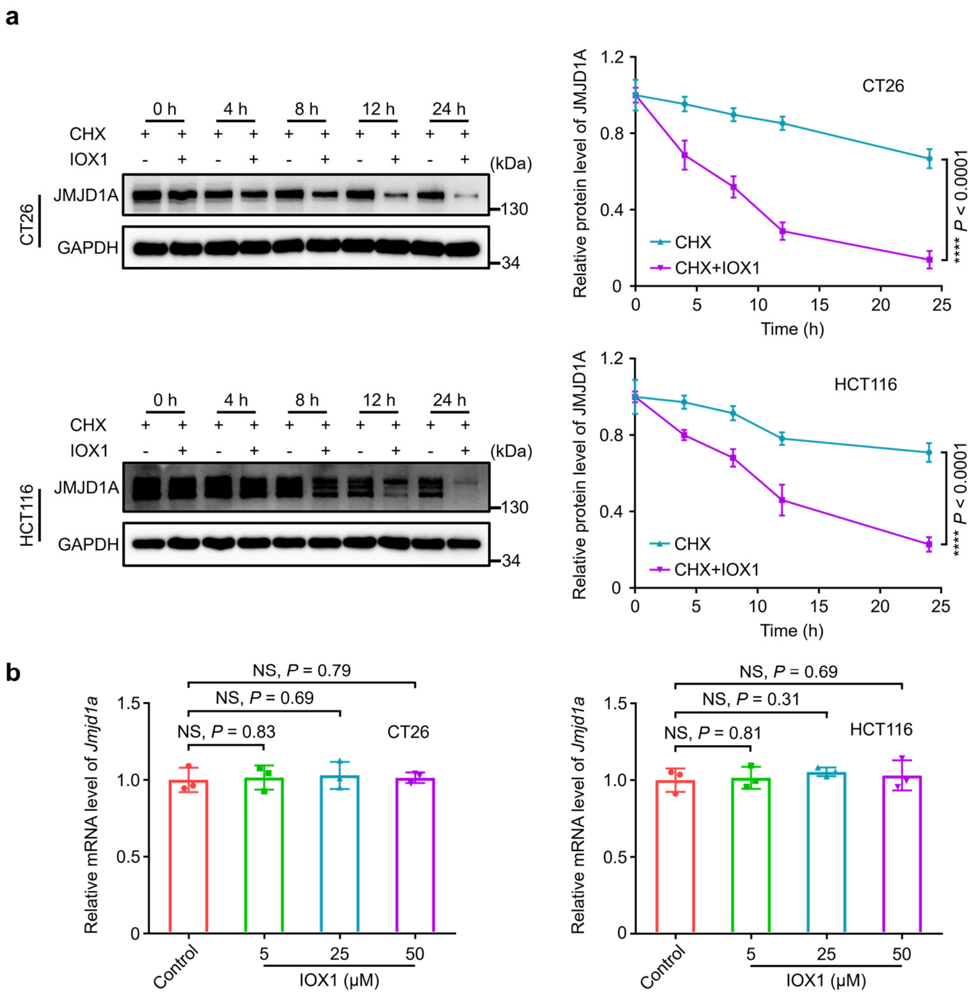

Supplementary Fig. 50. IOX1 treatment reduces the stability of JMJD1A protein. a, Western blotting images and their quantification of JMJD1A expression in CT26 or HCT116 cells at different times after treating with cycloheximide (CHX, 50 μg ml^{-1}) and IOX1 (50 μM) ; $n = 3$ independent experiments. **b,** The *Jmjd1a* mRNA levels of CT26 or HCT116 cells after treating with IOX1 (5, 25, or 50 μM) for 24 h analyzed by qPCR; $n = 3$ independent experiments. Data represent mean ± SD. Two-tailed Student's *t*-test. NS, no significance; **** $P < 0.0001$. Source data are provided as a Source Data file.

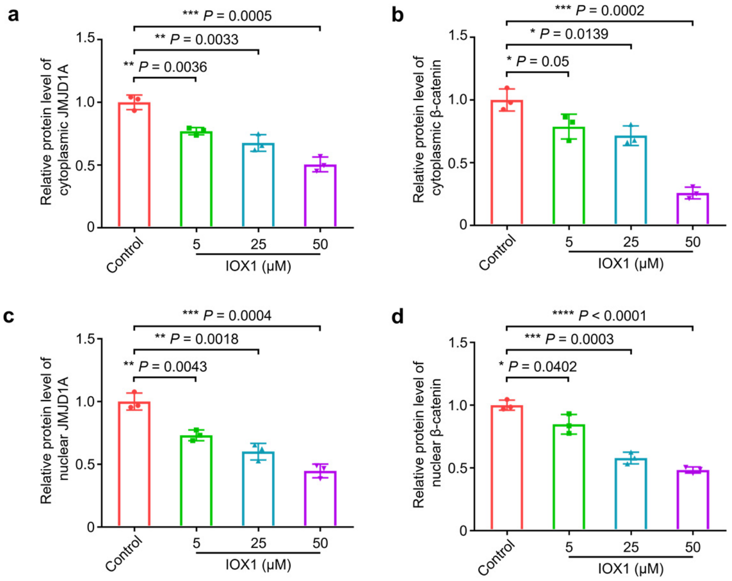

Supplementary Fig. 51. JMJD1A and β-catenin levels in IOX1-treated CT26 cells. Quantification by western blotting of **a,** cytoplasmic JMJD1A, **b,** cytoplasmic β-catenin, **c,** nuclear JMJD1A and **d,** nuclear β-catenin in CT26 cells after treating for 24 h with IOX1 (5, 25, or 50 μM). $n = 3$ independent experiments. Data represent mean ± SD. Two-tailed Student's *t*-test. * $P < 0.05$, ** $P < 0.01$, *** $P < 0.001$, **** $P < 0.0001$. See Fig. 4h in the text for images and other conditions. Source data are provided as a Source Data file.

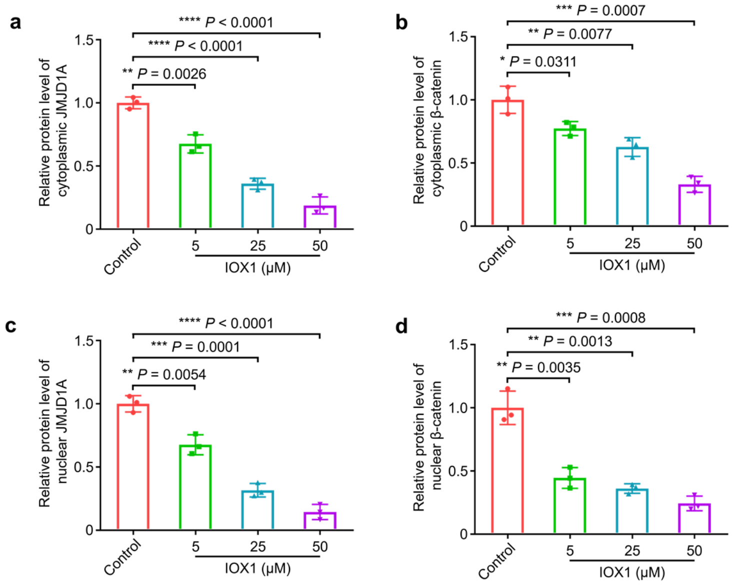

Supplementary Fig. 52. JMJD1A and β-catenin levels in IOX1-treated HCT116 cells. Quantification by western blotting of **a,** cytoplasmic JMJD1A, **b,** cytoplasmic β-catenin, **c,** nuclear JMJD1A and **d,** nuclear β-catenin in HCT116 cells after treating for 24 h with IOX1 (5, 25, or 50 μM). $n = 3$ independent experiments. Data represent mean ± SD. Two-tailed Student's *t*-test. * $P < 0.05$, ** $P < 0.01$, *** $P < 0.001$, **** $P < 0.0001$. See Fig. 4i in the text for images and other conditions. Source data are provided as a Source Data file.

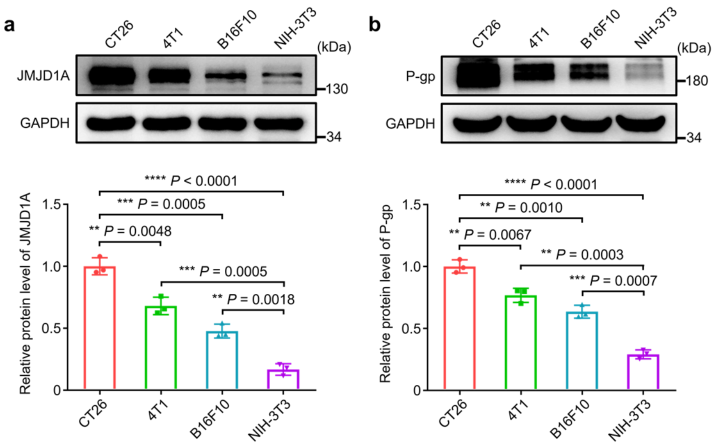

Supplementary Fig. 53. JMJD1A and P-gp levels in untreated murine cell lines. Western blotting images and their quantification of **a,** JMJD1A and **b,** P-gp expression. $n = 3$ independent experiments. Data represent mean ± SD. Two-tailed Student's t-test. ** $P < 0.01$, *** $P < 0.001$, **** $P < 0.0001$. Source data are provided as a Source Data file.

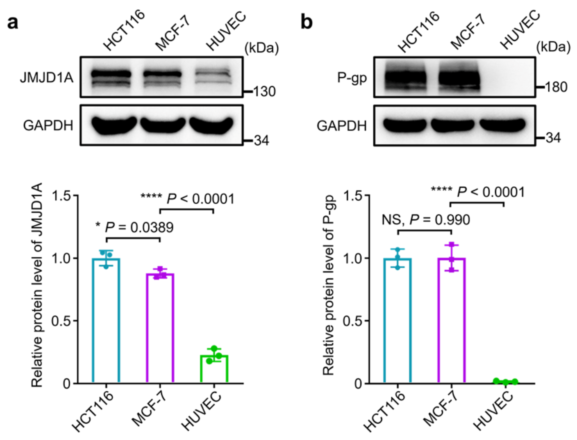

Supplementary Fig. 54. JMJD1A and P-gp levels in untreated human cell lines. Western blotting images and their quantification of **a,** JMJD1A and **b,** P-gp expression in human cell lines. $n = 3$ independent experiments. Data represent mean ± SD. Two-tailed Student's t-test. * $P < 0.05$, **** $P < 0.0001$. Source data are provided as a Source Data file.

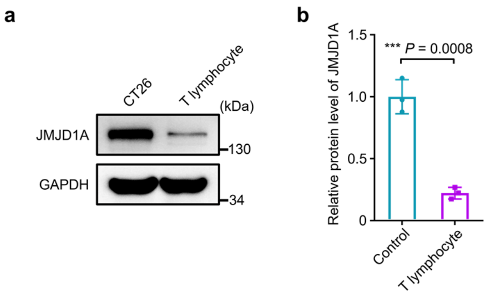

Supplementary Fig. 55. JMJD1A expression in untreated CT26 cells or T lymphocytes. a, Western blotting images and **b,** their quantification. $n = 3$ independent experiments. Data represent mean ± SD. Two-tailed Student's t-test. *** $P < 0.001$. Source data are provided as a Source Data file.

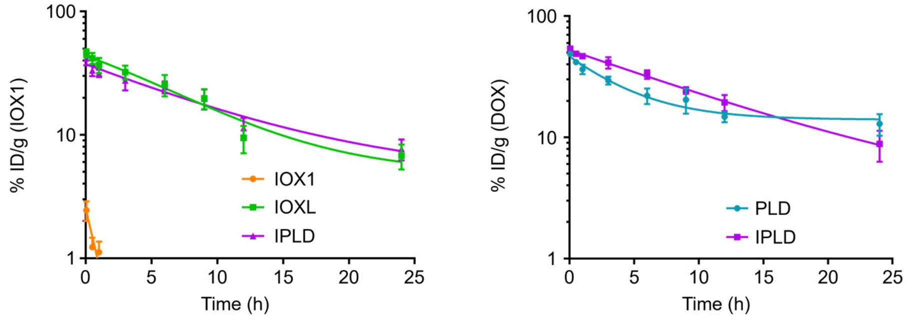

Supplementary Fig. 56. The blood clearance curves in BALB/c mice. Dose: IOX1-eq. 7.5 mg kg^{-1}, DOX-eq. 5 mg kg^{-1}; n = 3 mice. Data represent mean ± SD. Source data are provided as a Source Data file.

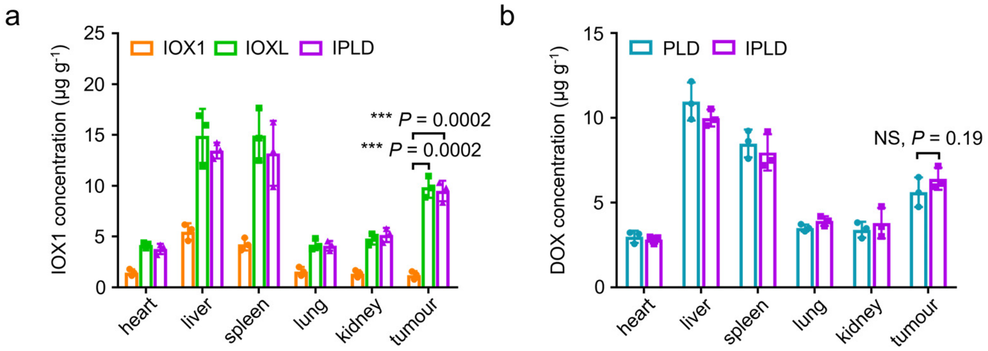

Supplementary Fig. 57. Biodistribution of IOX1 and DOX in s.c. CT26 tumour-bearing mice. a, The IOX1 and **b,** DOX concentrations in tumours and main organs of mice at 24 h post-intravenous injection of different formulations. Dose: IOX1-eq. 7.5 mg kg^{-1}, DOX-eq. 5 mg kg^{-1}; n = 3 mice. Data represent mean ± SD. Two-tailed Student's t-test. NS, no significance; *** $P < 0.001$. Source data are provided as a Source Data file.

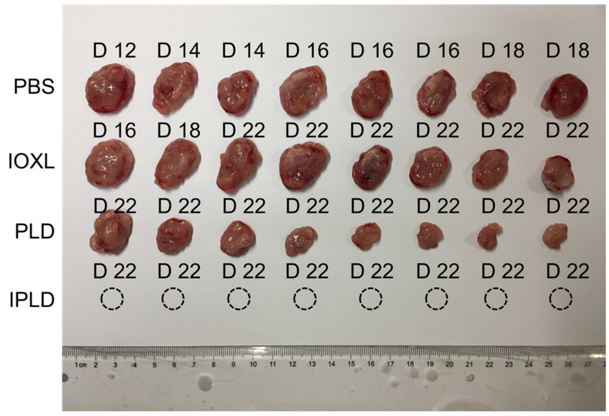

Supplementary Fig. 58. Images of the tumours from the treated s.c. 80 mm^3 CT26 tumour-bearing BALB/c mice in Figs. 5a-e. The tumours were harvested at the experimental endpoint (Day 22) or at the indicated days when reaching the allowed maximum.

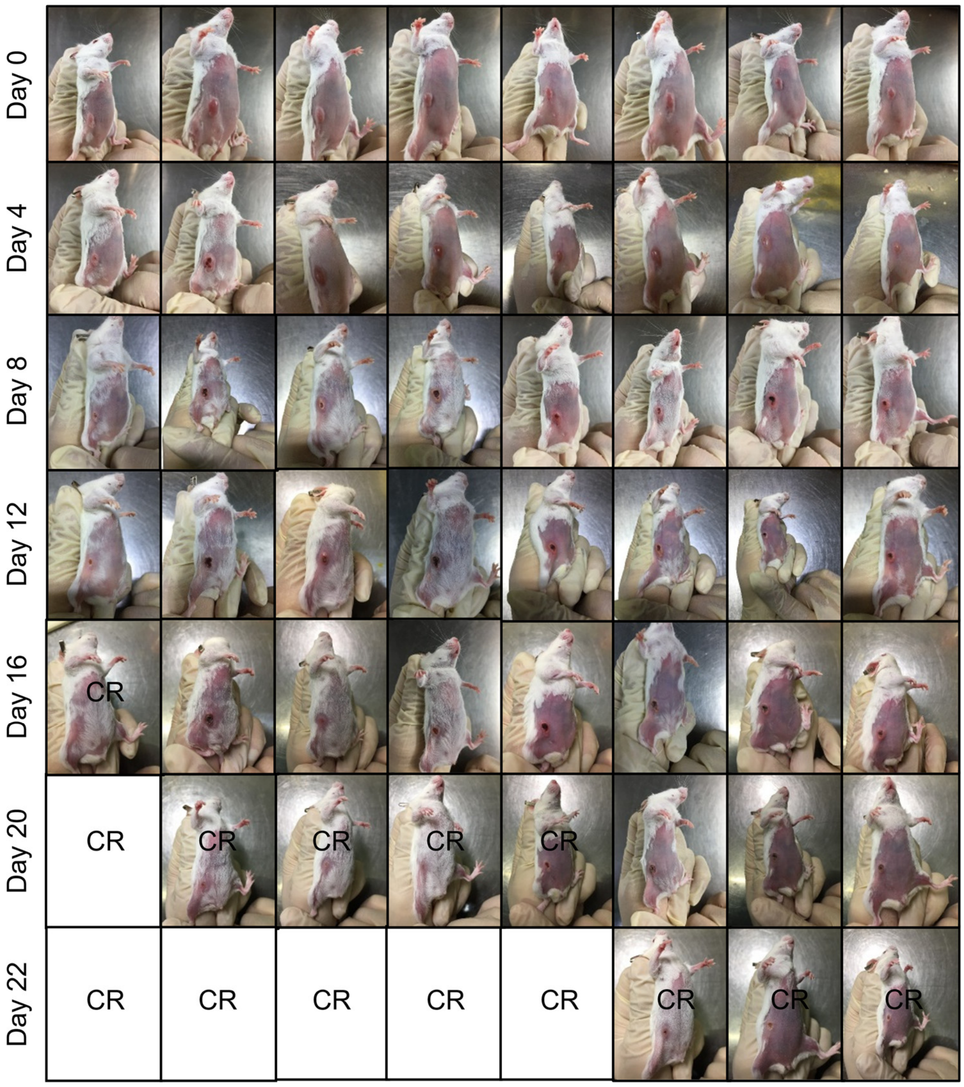

Supplementary Fig. 59. Photographs of the s.c. 80 mm^3 CT26 tumour-bearing BALB/c mice treated with IPLD during the experiment. CR: complete response. See Figs. 5a-g in the text for other conditions.

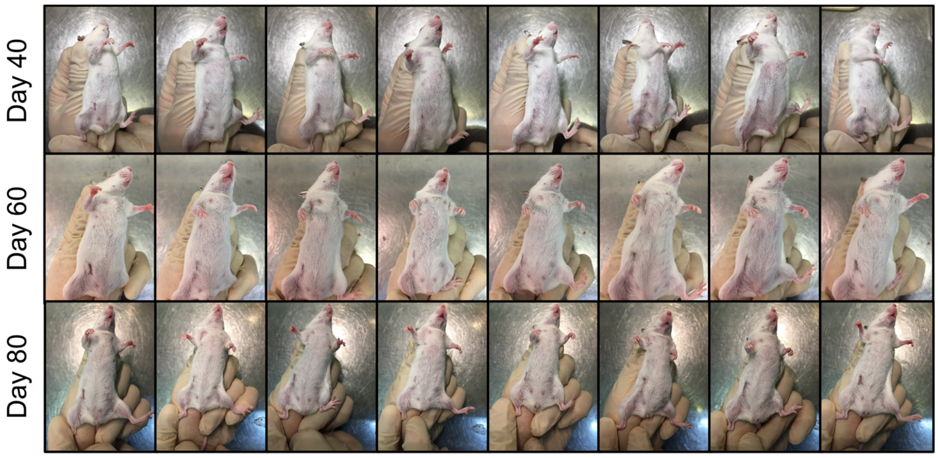

Supplementary Fig. 60. The follow-up tracking of the IPLD-treated s.c. 80 mm^3 CT26 tumour-bearing BALB/c mice. See Figs. 5a-g in the text for other conditions.

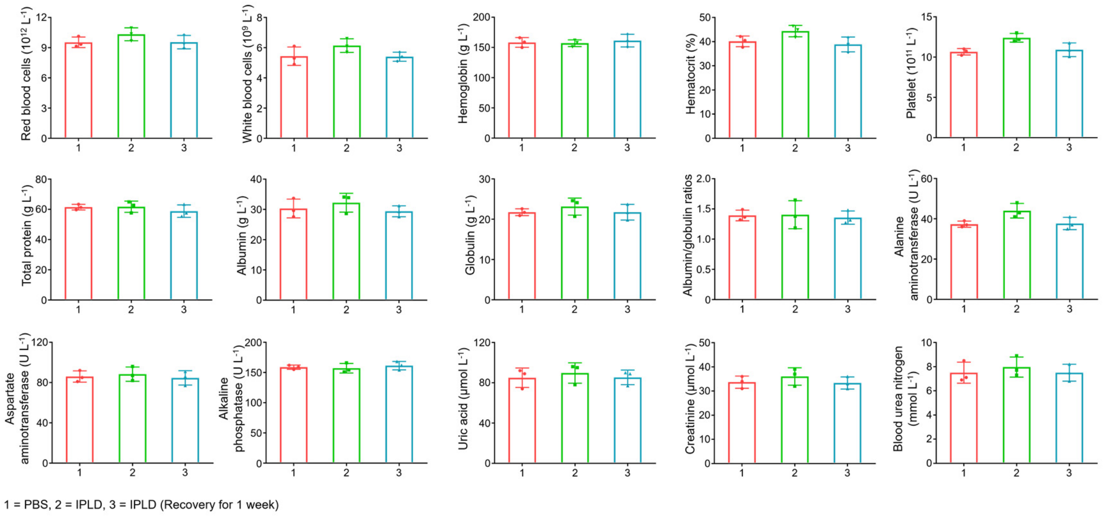

Supplementary Fig. 61. Blood biochemistry of the BALB/c mice after different treatments. Dose: IOX1-eq. 7.5 mg kg^{-1}, DOX-eq. 5 mg kg^{-1} for 4 times; The blood was collected on the day after the last treatment or 7 days post-recovery. $n = 3$ mice. Data represent mean ± SD. Source data are provided as a Source Data file.

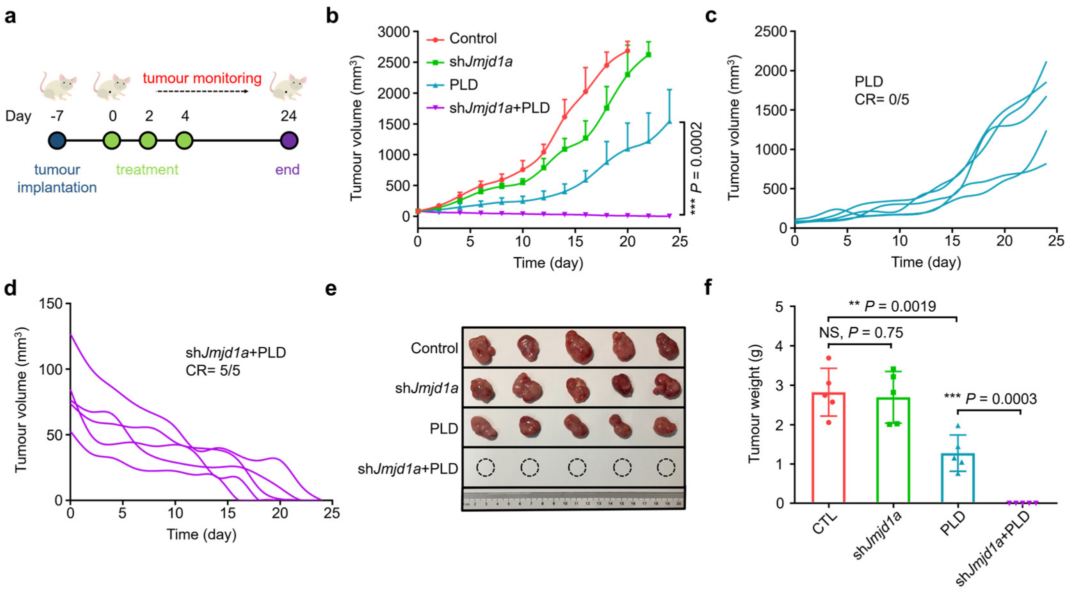

Supplementary Fig. 62. PLD treatment of s.c. JMJD1A-knockdown CT26 tumours. a, Experimental schedule; After 7 days of s.c. inoculation with 5×10^5 control or JMJD1A-knockdown CT26 cells, the tumours reached about 80 mm^3; the treatment was initiated (noted as Day 0) via the tail vein injection with PBS or PLD at 5 mg kg^{-1} DOX on every 2 days for 3 times. **b,** The tumour growth curves; $n = 5$ mice. **c,d,** The individual tumour growth curves of the PLD group or the shJMJD1A+PLD group in *b*; CR: complete response. **e,** The images of the tumours dissected at the end of experiment. **f,** The averaged tumour weight of each group on Day 24; $n = 5$ mice. Data represent mean ± SD. Two-tailed Student's *t*-test. NS, no significance; ** $P < 0.01$, *** $P < 0.001$. Source data are provided as a Source Data file.

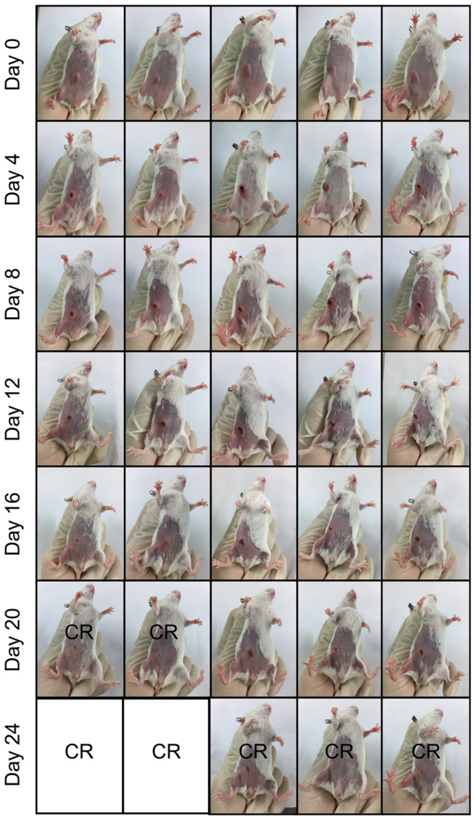

Supplementary Fig. 63. Photographs of the s.c. 80 mm³ JMJD1A-knockdown CT26 tumour-bearing BALB/c mice treated with PLD during the experiment. CR: complete response. See Supplementary Fig. 62 for other conditions.

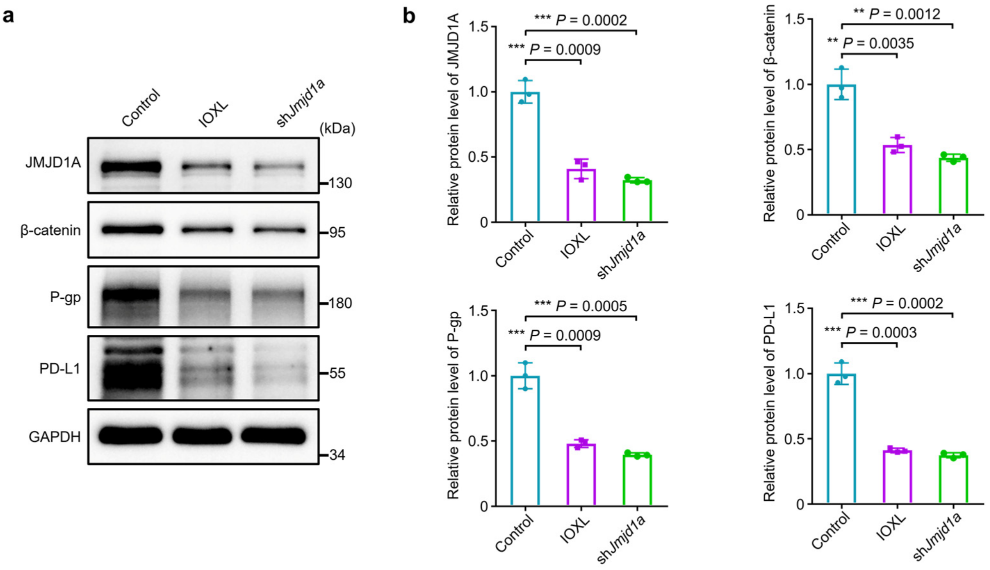

Supplementary Fig. 64. JMJD1A, β-catenin, P-gp and PD-L1 expression in the IOXL-treated CT26 tumours or JMJD1A-knockdown CT26 tumours. a, Western blotting images and **b,** their quantification; $n = 3$ mice. Data represent mean ± SD. Two-tailed Student's t-test. ** $P < 0.01$, *** $P < 0.001$. See Fig. 5a &Supplementary Fig. 62 for other conditions. Source data are provided as a Source Data file.

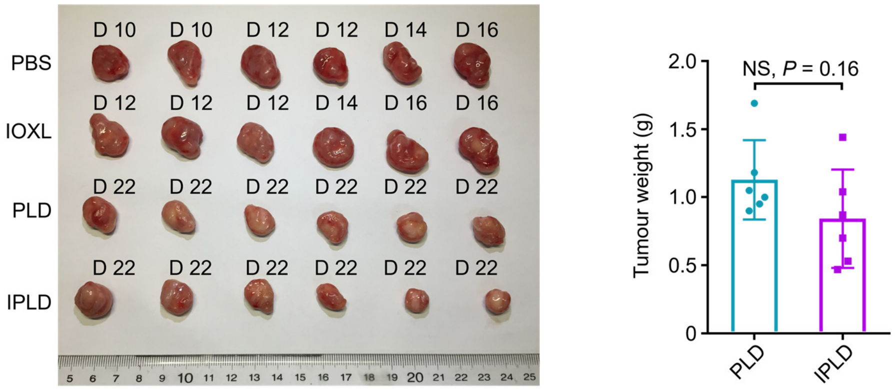

Supplementary Fig. 65. Images and the average tumour weights of the tumours from the treated s.c. 80 mm^3 CT26 tumour-bearing BALB/c nude mice in Fig. 5h. The tumours were harvested at the experimental endpoint (Day 22) or at the indicated days when reaching the allowed maximum. *n* = 6 mice. Two-tailed Student's t-test. NS, no significance. See Fig. 5h in the text for other conditions. Source data are provided as a Source Data file.

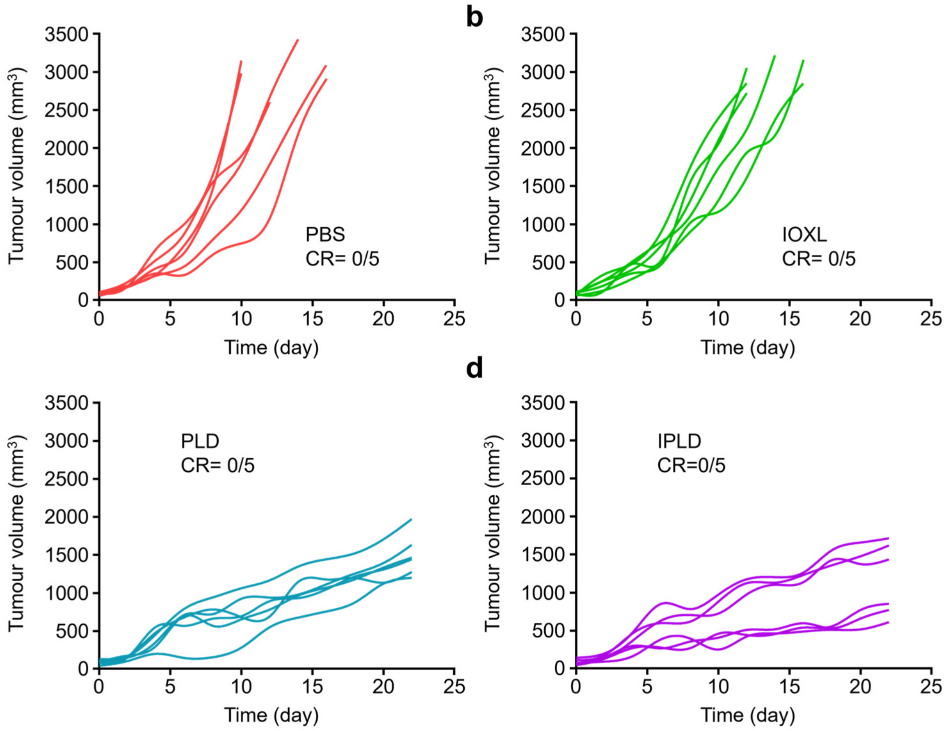

Supplementary Fig. 66. The individual tumour growth curves of the s.c. 80 mm^3 CT26 tumour-bearing nude mice after treatments. Mice were treated with **a,** PBS, **b,** IOXL (IOX1, 7.5 mg kg^{-1}) **c,** PLD (DOX, 5 mg kg^{-1}) or **d,** IPLD (IOX1, 7.5 mg kg^{-1}; DOX 5 mg kg^{-1}) for 3 times; ; CR: complete response. See Fig. 5h in the text for other conditions. Source data are provided as a Source Data file.

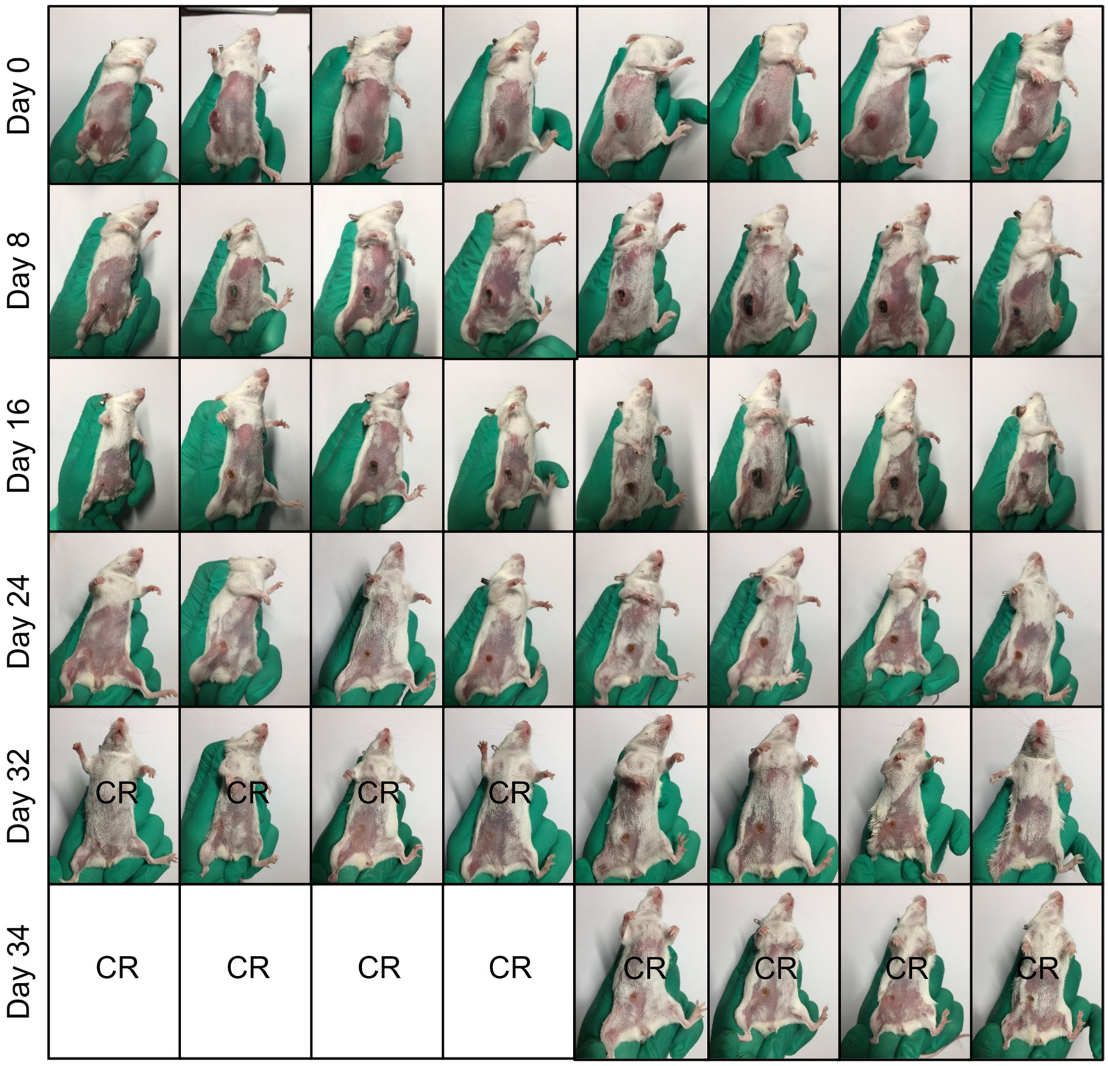

Supplementary Fig. 67. Photographs of the s.c. 350 mm³ CT26 tumour-bearing BALB/c mice treated with IPLD during the experiment. CR: complete response. See Figs. 5i-n in the text for other conditions.

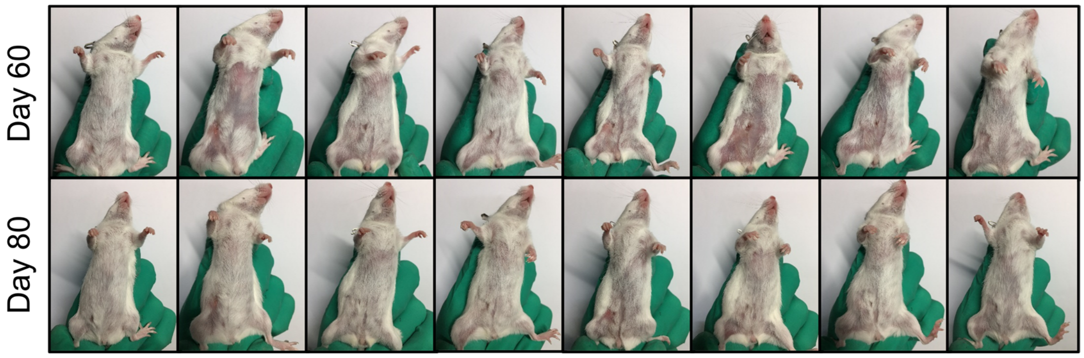

Supplementary Fig. 68. The follow-up tracking of the IPLD-treated s.c. 350 mm³ CT26 tumour-bearing BALB/c mice. See Figs. 5i-n in the text for other conditions.

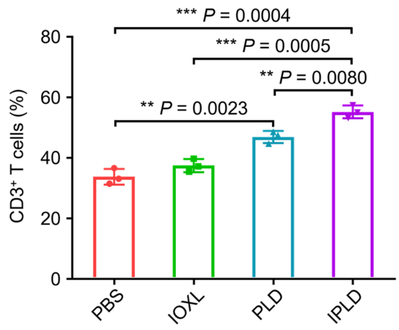

Supplementary Fig. 69. Flow cytometric quantification of intratumoural $CD3^+$ T cells in the treated s.c. 80 mm^3 CT26 tumour-bearing BALB/c mice. *n* = 3 mice. Data represent mean ± SD. Two-tailed Student's *t*-test. ** $P < 0.01$, *** $P < 0.001$. See Fig. 6 in the text for other conditions. Source data are provided as a Source Data file.

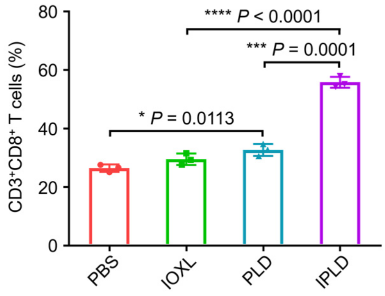

Supplementary Fig. 70. Flow cytometric quantification of splenocytes' $CD3^+CD8^+$ T cells in treated s.c. 80 mm^3 CT26 tumour-bearing BALB/c mice. *n* = 3 mice. Data represent mean ± SD. Two-tailed Student's *t*-test. * $P < 0.05$, *** $P < 0.001$, **** $P < 0.0001$. See Fig. 6m in the text for contour diagrams and other conditions. Source data are provided as a Source Data file.

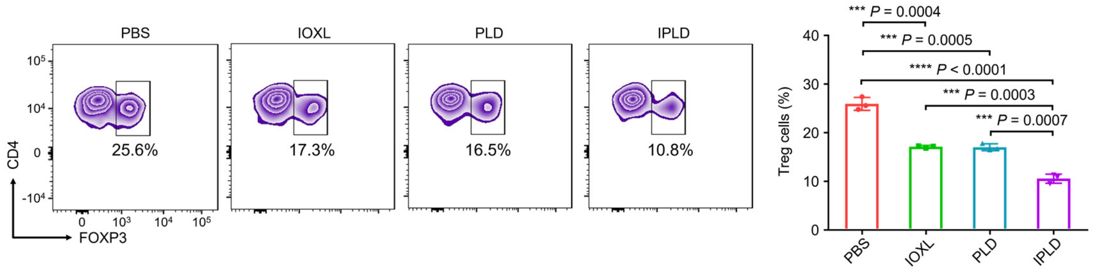

Supplementary Fig. 71. Flow cytometry analysis and quantification of splenocytes' Treg cells in the treated s.c. 80 mm^3 CT26 tumour-bearing BALB/c mice. *n* = 3 mice. Data represent mean ± SD. Two-tailed Student's *t*-test. *** $P < 0.001$, **** $P < 0.0001$. See Fig. 6 in the text for other conditions. Source data are provided as a Source Data file.

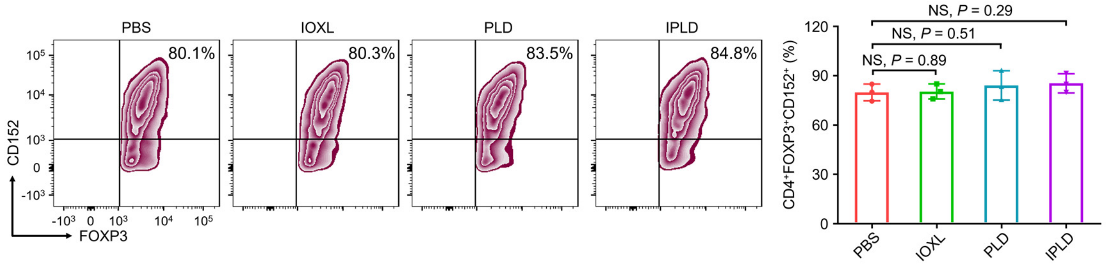

Supplementary Fig. 72. Flow cytometry analysis and quantification of CTLA-4 expression in intratumoural Treg cells in the treated s.c. 80 mm^3 CT26 tumour-bearing BALB/c mice. $n = 3$ mice. Two-tailed Student's t-test. NS, no significance. See Fig. 6 in the text for other conditions. Source data are provided as a Source Data file.

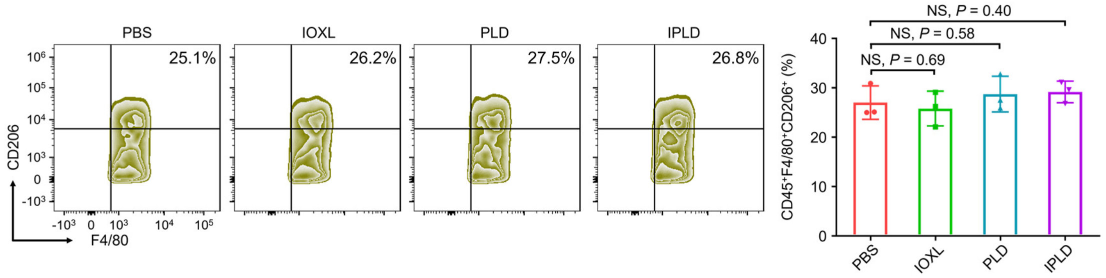

Supplementary Fig. 73. Flow cytometry analysis and quantification of tumour associated M2 macrophages in the treated s.c. 80 mm^3 CT26 tumour-bearing BALB/c mice. $n = 3$ mice. Two-tailed Student's t-test. NS, no significance. See Fig. 6 in the text for other conditions. Source data are provided as a Source Data file.

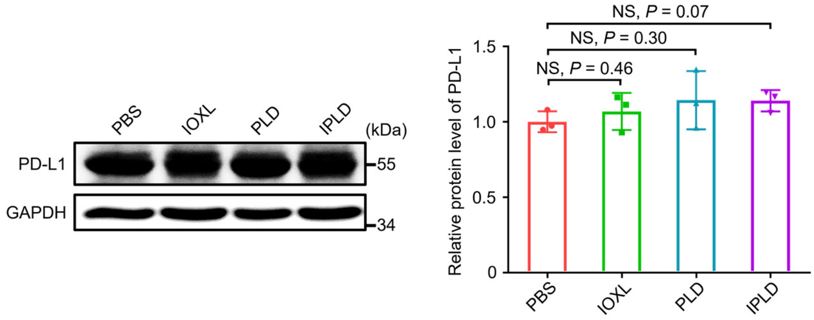

Supplementary Fig. 74. Western blotting images and their quantification of PD-L1 expression in pancreas in the s.c. 80 mm^3 CT26 tumour-bearing BALB/c mice after different treatments. $n = 3$ mice. Two-tailed Student's t-test. NS, no significance. See Fig. 6 in the text for other conditions. Source data are provided as a Source Data file.

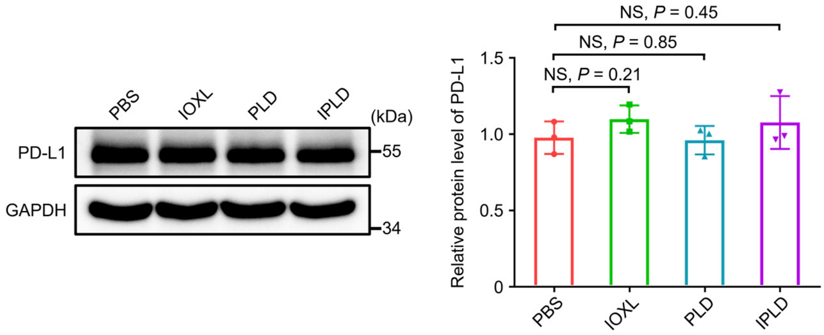

Supplementary Fig. 75. Western blotting images and their quantification of PD-L1 expression in lungs in the s.c. 80 mm^3 CT26 tumour-bearing BALB/c mice after different treatments. n = 3 mice. Two-tailed Student's t-test. NS, no significance. See Fig. 6 in the text for other conditions. Source data are provided as a Source Data file.

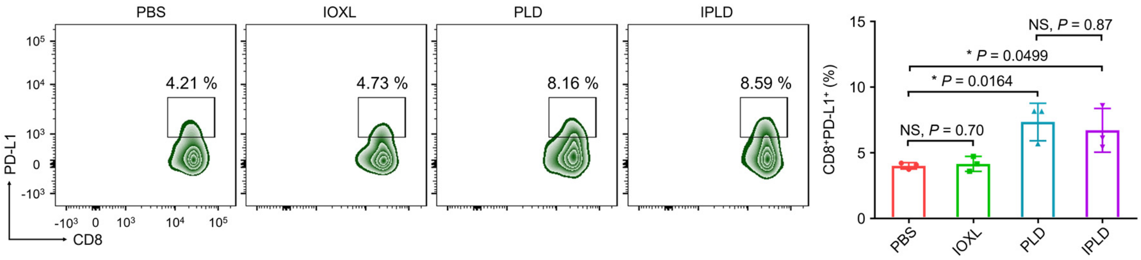

Supplementary Fig. 76. Flow cytometry analysis of intratumour CD8$^+$PD-L1$^+$ T cells in the s.c. 80 mm^3 CT26 tumour-bearing BALB/c mice after different treatments. n = 3 mice. Two-tailed Student's t-test. NS, not significant; * $P < 0.05$. See Fig. 6 in the text for other conditions. Source data are provided as a Source Data file.

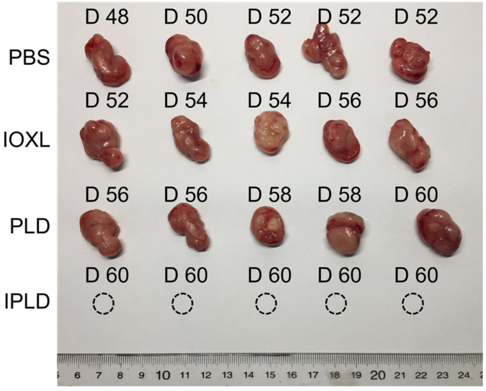

Supplementary Fig. 77. Images of the rechallenged tumours harvested on the indicated days or at the experimental endpoint (Day 60) from the s.c. CT26 rechallenged tumour-bearing BALB/c mice in Figs. 7a-c.

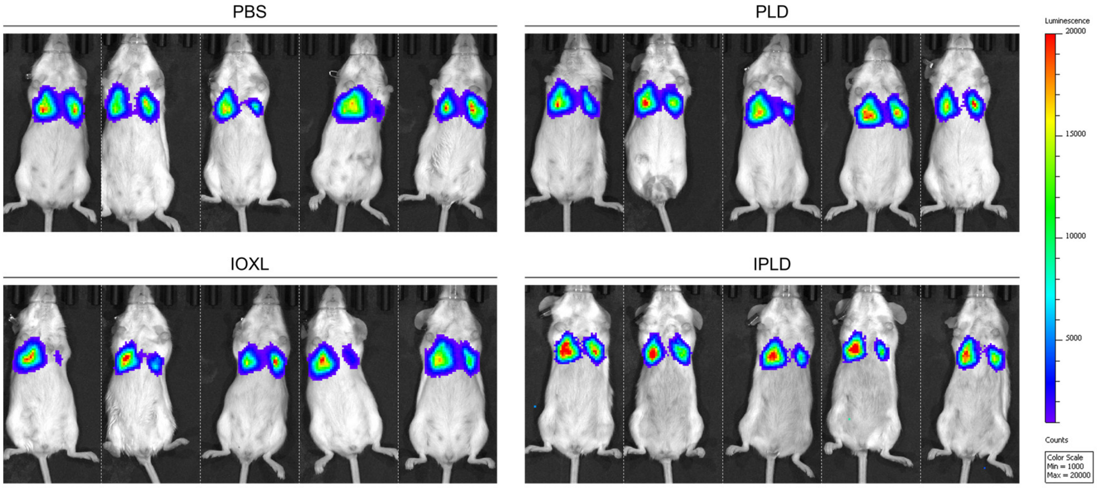

Supplementary Fig. 78. In vivo bioluminescence imaging of the lungs in anaesthetized mice immediately after i.v. injection of ^{Luci}CT26 cells. n = 5 mice. See Fig. 7e in the text for other conditions.

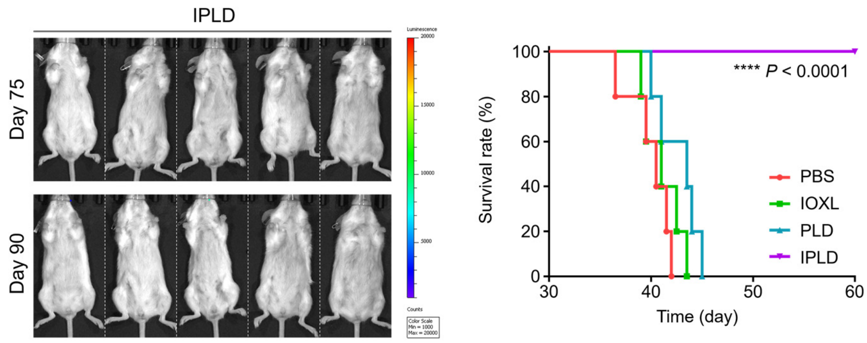

Supplementary Fig. 79. The follow-up tracking via in vivo bioluminescence imaging and the survival of the ^{Luci}CT26-rechallenged BALB/c mice. n = 5 mice. Statistical significance was analyzed by the log-rank (Mantel–Cox) test. **** $P < 0.0001$. See Fig. 7e in the text for other conditions. Source data are provided as a Source Data file.

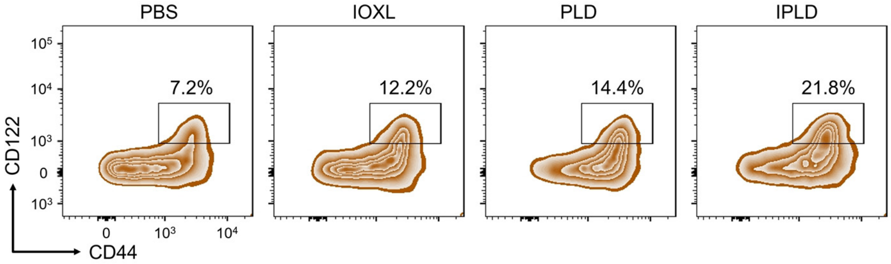

Supplementary Fig. 80. Flow cytometry analysis of splenocytes' $CD4^+CD44^+CD122^+$ T cells in the treated s.c. 80 mm^3 CT26 tumour-bearing BALB/c mice. The experiment was repeated independently three times to confirm the results. See Fig. 7d in the text for other conditions.

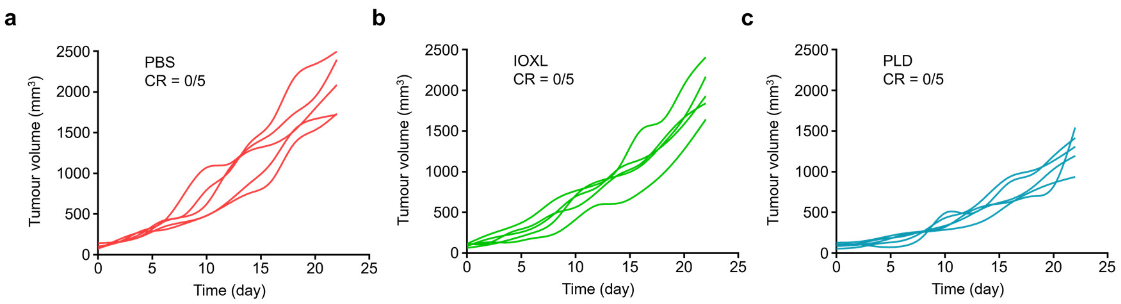

Supplementary Fig. 81. The individual tumour growth curves of the 4T1 orthotopic tumour-bearing BALB/c mice after treatments. a, PBS, **b,** IOXL (IOX1, 7.5 mg kg^{-1}) or **c,** PLD (DOX, 5 mg kg^{-1}) for 3 times; CR: complete response. See Figs. 8a,c in the text for other conditions. Source data are provided as a Source Data file.

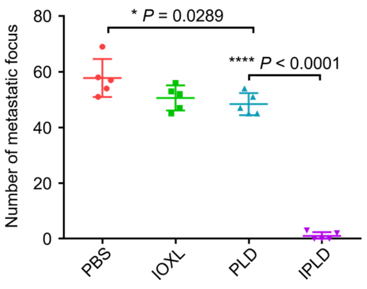

Supplementary Fig. 82. The average numbers of lung metastatic nodules in the treated dual 4T1 tumour-bearing BALB/c mice. n = 5 mice. Data represent mean ± SD. Two-tailed Student's t-test. * $P < 0.05$, **** $P < 0.0001$. See Fig. 8g in the text for images and other conditions. Source data are provided as a Source Data file.

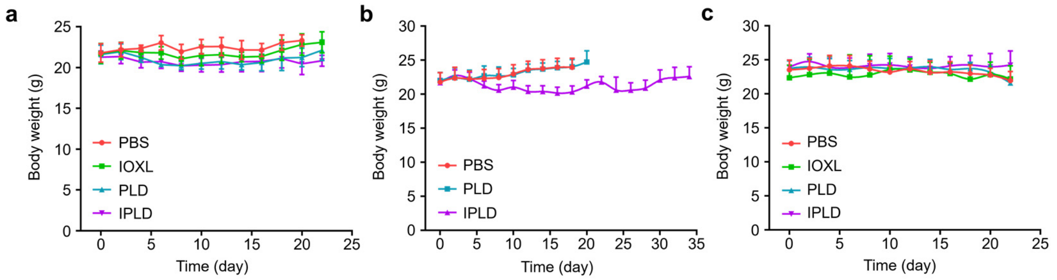

Supplementary Fig. 83. Body weight changes of the mice after treatments. a, s.c. 80 mm^3 CT26 tumours (n = 8 mice), **b,** s.c. 350 mm^3 CT26 tumours (n = 8 mice), or **c,** 4T1 orthotopic tumours (n = 5 mice) with different treatments in Figs. 5a,i and Fig. 8a. Data represent mean ± SD. Source data are provided as a Source Data file.

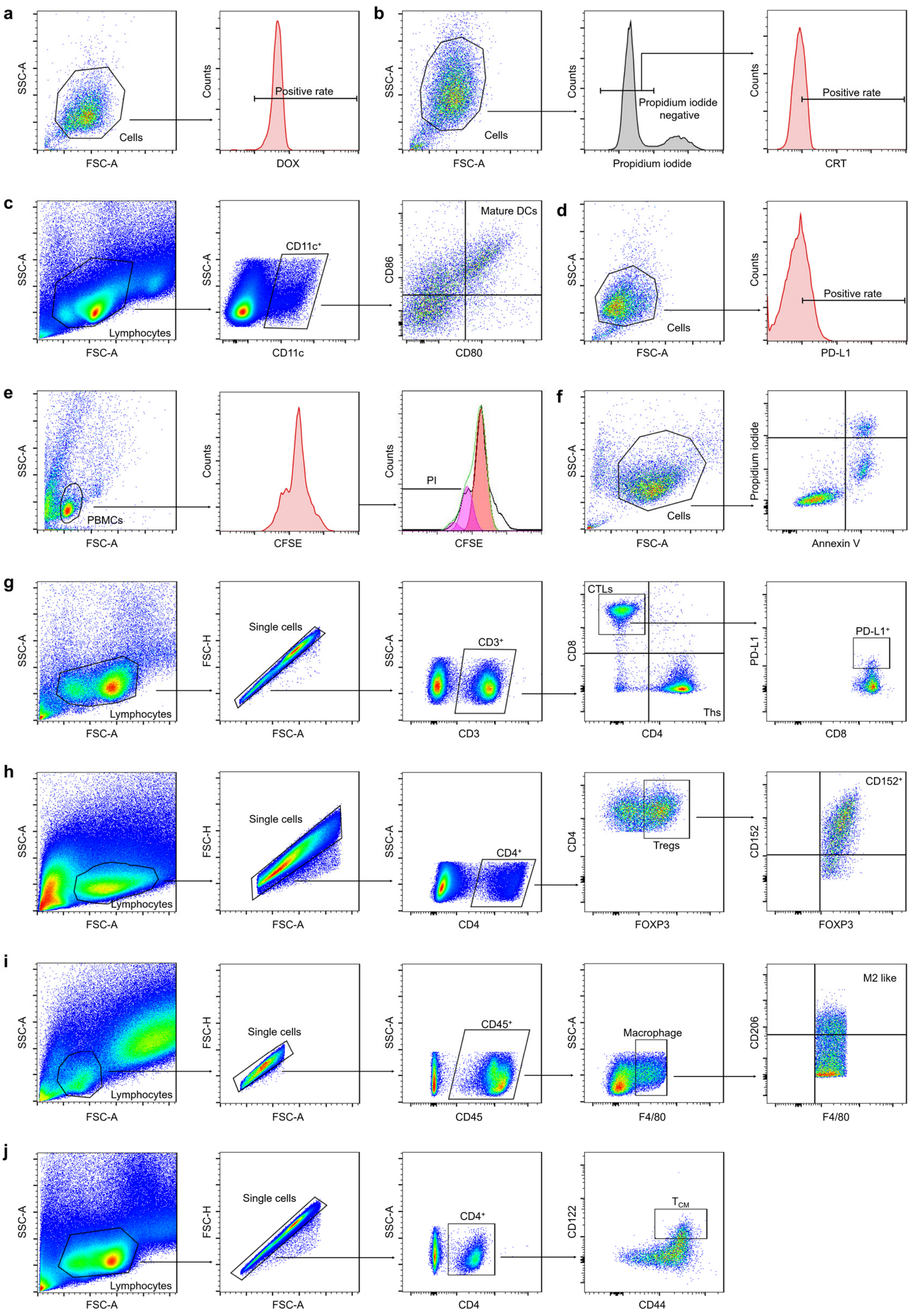

Supplementary Fig. 84. Gating strategies for flow cytometry. a, Gating strategy to determine DOX or rhodamine123 positive rate presented in Fig. 2e and Supplementary Figs. 9b,10b&d,11a, 12b&d,14b&d,15b&d,44a,45a. **b,** Gating strategy to determine CRT expression presented in Supplementary Figs. 19,23. **c,** Gating strategy to identify mature DCs presented on Figs. 3a,6d and Supplementary Figs. 27a,29a. **d,** Gating strategy to determine PD-L1 expression presented in Fig.

3d and Supplementary Figs. 31,33a,34,35a,46a,47a. **e,** Gating strategy to determine proliferation indices (PIs) of PBMC presented in Fig. 3g and Supplementary Figs. 37a,38a,40a. **f,** Gating strategy to determine apoptosis tumour cells presented on Fig. 3i and Supplementary Fig. 39a. **g,** Gating strategy to identify helper T cells ($CD3^+CD4^+$, Ths), cytotoxic T cells ($CD3^+CD8^+$, CTLs) and $CD8^+$PD-$L1^+$ T cells presented on Figs. 6f&m and Supplementary Fig. 76. **h,** Gating strategy to identify regulatory T cells ($CD4^+FOXP3^+$, Tregs) and $FOXP3^+CD152^+$ T cells presented on Fig. 6k and Supplementary Figs. 71,72. **i,** Gating strategy to identify tumour-associated M2 macrophages presented on Supplementary Fig. 73. **j,** Gating strategy to identify central memory T cells (T_{CM}) presented on Supplementary Fig. 80.

Supplementary Table 1. Primers (5'-3') used in this study.

Gene	Forward	Reverse
human *Jmjd1a*	CAGGAGCTCCACATCAGGTT	TGCATCTTTCACTGCATGGT
mouse *Jmjd1a*	TGAGTACACCAGGCGAGATG	GGTCCCATATTTCCGATCCT
human *Ctnnb1* (β-Catenin)	ACAAACTGTTTTGAAAATCCA	CGAGTCATTGCATACTGTCC
mouse *Ctnnb1* (β-Catenin)	TGGACCCCAAGCCTTAGTAAACA	GTCTGTCAGATGAAGCCCCAGTG
human *Cd274* (PD-L1)	ACAGCTGAATTGGTCATCCC	TGTCAGTGCTACACCAAGGC
mouse *Cd274* (PD-L1)	GACCAGCTTTTGAAGGGAAATG	CTGGTTGATTTTGCGGTATGG
human *Abcb1* (P-gp)	GACATCCCAGTGCTTCAGG	GCCACTGAACATTCAGTCG
mouse *Abcb1*(P-gp)	TACGACCCCATGGCTGGATC	GGTAGCGAGTCGATGAACTG
human *Gapdh*	AAGGTGAAGGTCGGAGTCAA	AATGAAGGGGTCATTGATGG
mouse *Gapdh*	GCATTGCCCTCAACGACCAC	CCACCACCCTGTTGCTGTAG
