# Supplementary material for: Co-delivery of IOX1 and doxorubicin for antibody-independent cancer chemo-immunotherapy
Source: Nat Commun. 2021 Apr 23;12:2425. doi: 10.1038/s41467-021-22407-6 (PMC8065121; doi:10.1038/s41467-021-22407-6)
Supplement: Supplementary file 3 — Reporting Summary [file 41467_2021_22407_MOESM3_ESM.pdf]

nature research

Corresponding author(s): Youqing Shen

Last updated by author(s): Mar 2, 2021

Reporting Summary

Nature Research wishes to improve the reproducibility of the work that we publish. This form provides structure for consistency and transparency in reporting. For further information on Nature Research policies, see our Editorial Policies and the Editorial Policy Checklist.

Statistics

For all statistical analyses, confirm that the following items are present in the figure legend, table legend, main text, or Methods section.

n/a	Confirmed	
☐	☒	The exact sample size (n) for each experimental group/condition, given as a discrete number and unit of measurement
☐	☒	A statement on whether measurements were taken from distinct samples or whether the same sample was measured repeatedly
☐	☒	The statistical test(s) used AND whether they are one- or two-sided<br>*Only common tests should be described solely by name; describe more complex techniques in the Methods section.*
☐	☒	A description of all covariates tested
☐	☒	A description of any assumptions or corrections, such as tests of normality and adjustment for multiple comparisons
☐	☒	A full description of the statistical parameters including central tendency (e.g. means) or other basic estimates (e.g. regression coefficient) AND variation (e.g. standard deviation) or associated estimates of uncertainty (e.g. confidence intervals)
☐	☒	For null hypothesis testing, the test statistic (e.g. F, t, r) with confidence intervals, effect sizes, degrees of freedom and P value noted<br>*Give P values as exact values whenever suitable.*
☒	☐	For Bayesian analysis, information on the choice of priors and Markov chain Monte Carlo settings
☒	☐	For hierarchical and complex designs, identification of the appropriate level for tests and full reporting of outcomes
☒	☐	Estimates of effect sizes (e.g. Cohen's d, Pearson's r), indicating how they were calculated

Our web collection on statistics for biologists contains articles on many of the points above.

Software and code

Policy information about availability of computer code

Data collection	Zetasizer software 7.02, NIS-Elements AR 3.2 software, BD CellQuest™ Pro Software, softMax Pro V5.4, Living Image® 4.5.2
Data analysis	All statistical analyses were analyzed by Microsoft office Excel 2016, Graphpad Prism 7. The western blotting images were analyzed by Image J 1.52a. Flow cytometry data were analyzed by FlowJo V 4.5. CLSM images were analyzed by NIS-Elements AR 3.2 software. Living Image® 4.5.2 (Perkin Elmer) was used to analyze bioluminescent images.

For manuscripts utilizing custom algorithms or software that are central to the research but not yet described in published literature, software must be made available to editors and reviewers. We strongly encourage code deposition in a community repository (e.g. GitHub). See the Nature Research guidelines for submitting code & software for further information.

Data

Policy information about availability of data

All manuscripts must include a data availability statement. This statement should provide the following information, where applicable:

- Accession codes, unique identifiers, or web links for publicly available datasets
- A list of figures that have associated raw data
- A description of any restrictions on data availability

The authors declare that the main data supporting the findings of this study are available within the Article and its Supplementary Information or from the authors upon reasonable request. Source data are provided with this paper.

Field-specific reporting

Please select the one below that is the best fit for your research. If you are not sure, read the appropriate sections before making your selection.

- [x] Life sciences
- [ ] Behavioural & social sciences
- [ ] Ecological, evolutionary & environmental sciences

For a reference copy of the document with all sections, see nature.com/documents/nr-reporting-summary-flat.pdf

Life sciences study design

All studies must disclose on these points even when the disclosure is negative.

Sample size	The sample sizes of this study were determined on the basis of similar published studies (Zhou, et al. Nat. Nanotechnol. 14, 799-809 (2019)). For the in vivo experiments we aimed for a number of at least 5 animals per group to allow basic statistical inference. For other experiments, the sample size for each group was 3-6. Details regarding sample size of all experiments are provided in the Methods section and figure legends.
Data exclusions	No data were excluded.
Replication	Experiments were repeated and experimental findings were reproducible. Details of experimental replicates are given in the figure legends. All reported attempts at replication were successful.
Randomization	The experimental groups were allocated randomly
Blinding	Animal treatments were performed by technicians who were not blind, but not involved in sample measurement. In in vitro experiments, blinding was not necessary since all the samples were analyzed in the same way. Analysis of all the data were performed by an investigator blinded to the experimental conditions.

Reporting for specific materials, systems and methods

We require information from authors about some types of materials, experimental systems and methods used in many studies. Here, indicate whether each material, system or method listed is relevant to your study. If you are not sure if a list item applies to your research, read the appropriate section before selecting a response.

Materials & experimental systems

n/a	Involved in the study	
[ ]	[x]	Antibodies
[ ]	[x]	Eukaryotic cell lines
[x]	[ ]	Palaeontology and archaeology
[ ]	[x]	Animals and other organisms
[x]	[ ]	Human research participants
[x]	[ ]	Clinical data
[x]	[ ]	Dual use research of concern

Methods

n/a	Involved in the study	
[x]	[ ]	ChIP-seq
[ ]	[x]	Flow cytometry
[x]	[ ]	MRI-based neuroimaging

Antibodies

Antibodies used

The following primary antibodies were used for western blotting. They are listed as antigen first, followed by supplier, catalog number clone/lot number as applicable.
1) Anti JMJD1A, Abcam cat.no.: ab106456, Dilution: 1:1000
2) Anti β-catenin, Abcam cat.no.: ab6302, Dilution: 1:4000
3) Anti mouse PD-L1, Abcam cat.no.: ab213480, Dilution: 1:1000
4) Anti human PD-L1, Abcam cat.no.: ab213524, Dilution: 1:1000
5) Anti P-gp, Abcam cat.no.: ab170904, Dilution: 1:1000
6) Anti calreticulin, Abcam cat.no.: ab92516, Dilution: 1:1000
7) Anti pan-Cadherin, Abcam cat.no.: ab51034, Dilution: 1:10000
8) Anti β-Actin, Abcam cat.no.: ab8226, Dilution: 1:1000
9) Anti GAPDH, Abcam cat.no.: ab181602, Dilution: 1:10000
10) Anti p-PERK, CST cat.no.: #3179, Dilution: 1:1000
11) Anti eIF2α, CST cat.no.: #5324, Dilution: 1:1000
12) Anti p-eIF2α, CST cat.no.: #3398, Dilution: 1:1000
13) HRP labeled goat anti-rabbit antibody, Beyotime, cat.no.: A0208, Dilution: 1:1000;
14) HRP labeled goat anti-mouse antibody, Beyotime, cat.no.: A0216, Dilution: 1:1000;
The following primary antibodies were used for flow cytometry and immunofluorescence. They are listed as antigen first, followed by supplier, catalog number and clone/lot number as applicable.

	1) Anti mouse PD-L1, Abcam cat.no.: ab213480, Dilution: 1:100.<br>2) Anti human PD-L1, Abcam cat.no.: ab213524, Dilution: 1:100.<br>3) Anti-Calreticulin, Abcam cat.no.: ab92516, Dilution:1:100.<br>4) Anti-HMGB1, Abcam cat.no.: ab79823, Dilution:1:100.<br>5) Anti-LC3B, Abcam cat.no.: ab48394, Dilution:1:200.<br>6) Anti-Granzyme B, Abcam cat.no.: ab4059, Dilution:1:50.<br>7) Alexa Fluor 647 conjugated goat anti-rabbit IgG antibody, Beyotime, cat.no.: A0468; Dilution: 1:200.<br>8) Alexa Fluor 488 conjugated goat anti-rabbit IgG antibody, Beyotime, cat.no.: A0423; Dilution: 1:200.<br>9) FITC anti-CD11c, Biolegend, cat.no.: 117305; Dilution: 1:250.<br>10) PE anti-CD80, Biolegend, cat.no.: 104707; Dilution: 1:100.<br>11) APC anti-CD86, Biolegend, cat.no.: 105011; Dilution: 1:50.<br>12) PerCP/Cy5.5 anti-CD44, Biolegend, cat.no.: 103032; Dilution: 1:100.<br>13) APC anti-CD122, Biolegend, cat.no.: 105911; Dilution: 1:100.<br>14) FITC anti-CD45, Biolegend, cat.no.: 103107; Dilution: 1:200.<br>15) APC anti-F4/80, Biolegend, cat.no.: 123115; Dilution: 1:100.<br>16) PE-Cy7-CD206, Biolegend, cat.no.: 141719; Dilution: 1:100.<br>17) PE-Cy7 anti-CD152, Biolegend, cat.no.: 106314; Dilution: 1:100.<br>18) APC anti-CD3, BD Biosciences, cat.no.: 561826; Dilution: 1:400.<br>19) FITC anti-CD8, BD Biosciences, cat.no.: 561966; Dilution: 1:50.<br>20) PE anti-CD4, BD Biosciences, cat.no.: 561829; Dilution: 1:200.<br>21) Alexa Fluor 647 anti-FOXP3, BD Biosciences, cat.no.: 560402; Dilution: 1:100.
Validation	All antibodies were validated by the manufacturers.

Eukaryotic cell lines

Policy information about cell lines

Cell line source(s)	Murine colon cancer cell line CT26 (ATCC® CRL-2638), murine breast cancer cell line 4T1 (ATCC® CRL-3406), murine melanoma cell line B16F10 (ATCC® CRL-6475), murine embryonic fibroblast cell line NIH-3T3 (ATCC® CRL-1658), human colon cancer cell line HCT116 (ATCC® CCL-247), human breast cancer cell line MCF-7 (ATCC® HTB-22) and human umbilical vein endothelial cell line HUVEC (ATCC® CRL-1730) were purchased from ATCC (American Type Culture Collection).
Authentication	Purchased from ATCC. All cells used were authenticated using STR profiling.
Mycoplasma contamination	The cell lines regularly tested for mycoplasma contamination, and no mycoplasma contamination was found.
Commonly misidentified lines<br>(See ICLAC register)	No commonly misidentified cell lines were used.

Animals and other organisms

Policy information about studies involving animals; ARRIVE guidelines recommended for reporting animal research

Laboratory animals	BALB/c mice (six weeks, female) and BALB/c nude (six weeks, female) mice were purchased from the Institute of Medicine of Zhejiang Province. Mice were housed in an animal facility under constant environmental conditions (room temperature, 21±1°C; relative humidity, 40–70%, and a 12-h light-dark cycle). All mice had access to food and water ad libitum.
Wild animals	The study did not involve wild animals.
Field-collected samples	The study did not involve samples collected from field.
Ethics oversight	All performances on animals were approved by the Animal Care and Use Committee of Zhejiang University as per the Chinese Guidelines for The Care and Use of Laboratory Animals.

Note that full information on the approval of the study protocol must also be provided in the manuscript.

Flow Cytometry

Plots

Confirm that:

- [x] The axis labels state the marker and fluorochrome used (e.g. CD4-FITC).
- [x] The axis scales are clearly visible. Include numbers along axes only for bottom left plot of group (a 'group' is an analysis of identical markers).
- [x] All plots are contour plots with outliers or pseudocolor plots.
- [x] A numerical value for number of cells or percentage (with statistics) is provided.

Methodology

Sample preparation	Bone marrow-derived dendritic cells (BMDCs) were extracted from the bone marrow of six-week-old female BALB/c mice and cultured in 1640 medium (2 ml) with GM-CSF (20 ng/ml) and IL-4 (10 ng/ml). Peripheral blood mononuclear cells (PBMCs) were obtained from the blood of BALB/c mice by density gradient centrifugation. Cultured cells were trypsin digested and washed with PBS for twice, then collected for antibodies staining and flow cytometric analysis. For cellular uptake study, the cells were collected and directly analyzed by flow cytometry.<br>For in vivo antitumour immune response study, the mice were euthanasia and their tumours, spleens or tumour-draining lymph nodes (TDLNs) were harvested. The tissues were cut into small pieces and immersed in the solution of 1 mg/ml collagenase IVand 0.2 mg /ml DNase I at 37°C for 1 h. The mixtures were filtered to obtain single-cell suspension solutions. The cells were washed with PBS and stained with fluorescent labeled antibodies against specific cell markers (see detail in Methods section). Then samples were washed and subjected to flow cytometric analysis.
Instrument	The data were acquired using FACSCalibur™(Becton Dickinson, San Jose, USA).
Software	Data acquired by FACS Calibur™ was analyzed using BD CellQuest™ Pro Software (BD Biosciences) and FlowJo software package.
Cell population abundance	The number of cells sorting in the target gate was at least 5000.
Gating strategy	Cells were gated by FSC/SSC gates and then FSC/FSC-Height to select single cells. Other detail gating strategy was showed as follows:<br>Mature DCs: CD11c+, CD80+, CD86+;<br>Cytotoxic T cells (CTLs): CD3+, CD4-, CD8+ (PD-L1 expression was examined);<br>Helper T cells (Ths): CD3+, CD4+, CD8-;<br>Regulatory T cells (Tregs): CD4+, FOXP3+ (CD152 expression was examined);<br>Central memory T cells (TCM): CD4+, CD44+, CD122+;<br>Tumour-associated M2 macrophages: CD45+, F4/80+, CD206+.

☒ Tick this box to confirm that a figure exemplifying the gating strategy is provided in the Supplementary Information.
